# Supplementary material for: TK216 targets microtubules in Ewing sarcoma cells
Source: Cell Chem Biol. 2022 Aug 18;29(8):1325–1332.e4. doi: 10.1016/j.chembiol.2022.06.002 (PMC9394687; doi:10.1016/j.chembiol.2022.06.002)
Supplement: Document S1. Figures S1–S4 and Data S1 and S2 [file mmc1.pdf]

Cell Chemical Biology, Volume *29*

Supplemental information

TK216 targets microtubules

in Ewing sarcoma cells

Juan Manuel Povedano, Vicky Li, Katherine E. Lake, Xin Bai, Rameshu Rallabandi, Jiwoong Kim, Yang Xie, Jef K. De Brabander, and David G. McFadden

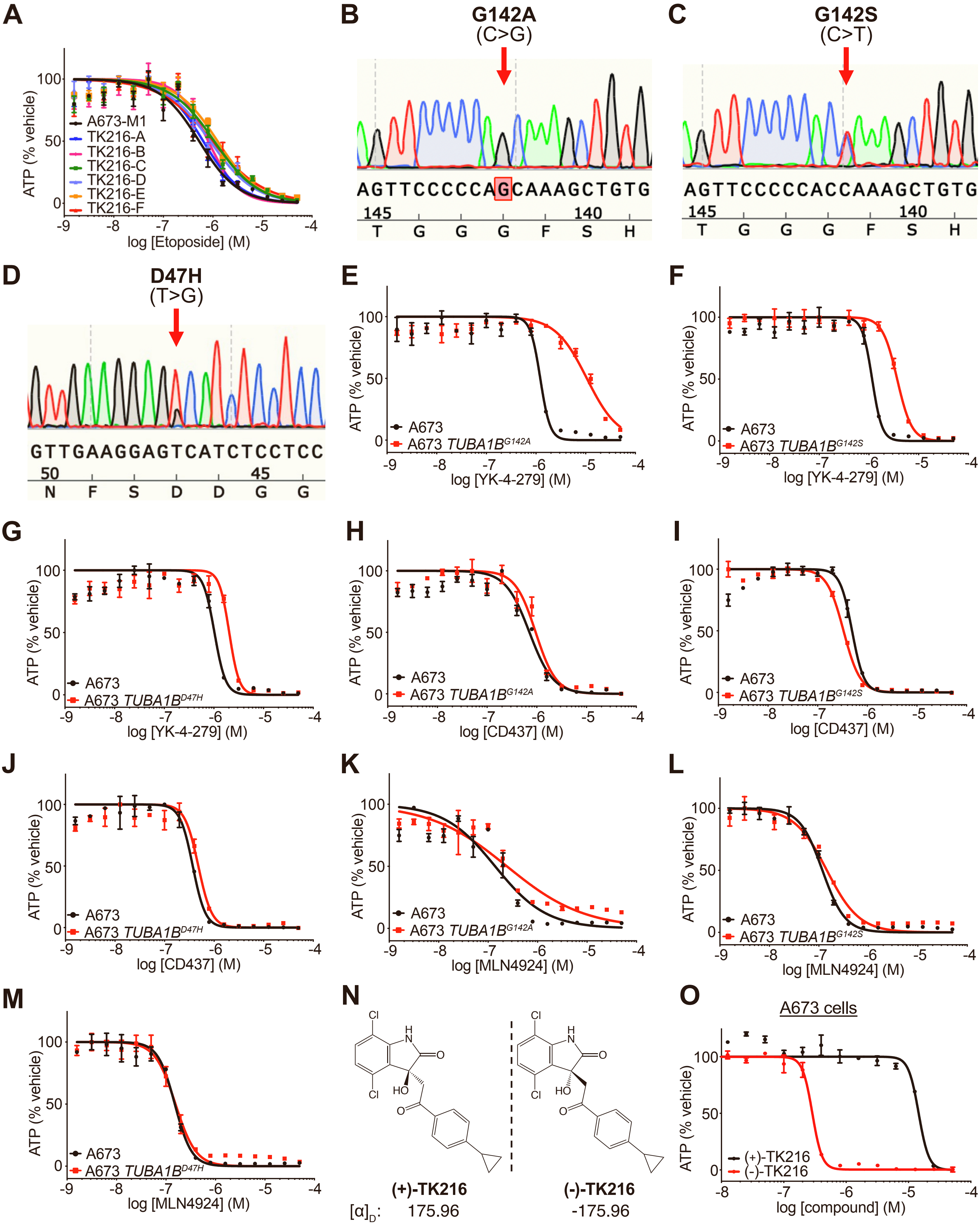

Figure S1, related to Figure 1. (A) Dose-response curve for etoposide against A673-M1, Msh2-null EWS cells, and TK216 resistant clones. (B,C,D) Sanger sequencing traces for *TUBA1B*^{G142A}, *TUBA1B*^{G142S}, and *TUBA1B*^{D47H} mutations in EWS cells. (E,H,K) Dose-response curves for, YK-4-279 (E), CD437 (H), and MLN4924 (K) against EWS cells harboring *TUBA1B*^{G142A} mutation. (F,I,L) Dose-response curves for, YK-4-279 (F), CD437 (I), and MLN4924 (L) against EWS cells harboring *TUBA1B*^{G142S} mutation. (G,J,M) Dose-response curves for, YK-4-279 (G), CD437 (J), and MLN4924 (M) against EWS cells harboring *TUBA1B*^{D47H} mutation. (N) Chemical structure and specific rotation ($[\alpha]_D$) of each TK216 enantiomer. (O) Dose-response curve for (-)-TK216 and (+)-TK216, against EWS A673 cells.

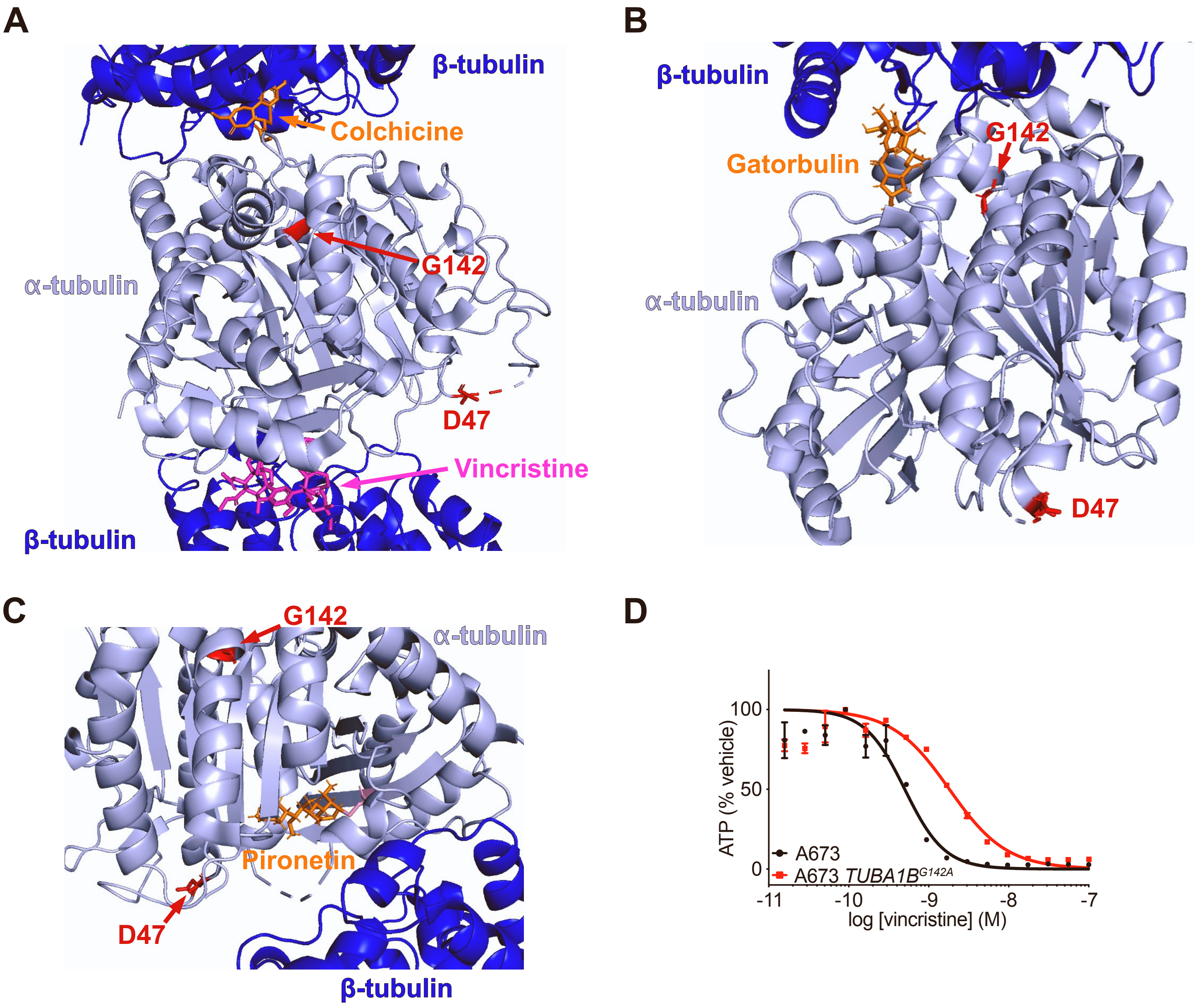

Figure S2, related to Figures 1 and 2. (A-C) Co-crystal structure of α-/β-tubulin with colchicine and vincristine (PDB: 1Z2B) (A), gatorbulin (PDB: 7alr) (B), and pironetin (PDB: 5la6) (C). (D) Dose-response curve for vincristine against EWS cells harboring *TUBA1B*^{G142A} mutation.

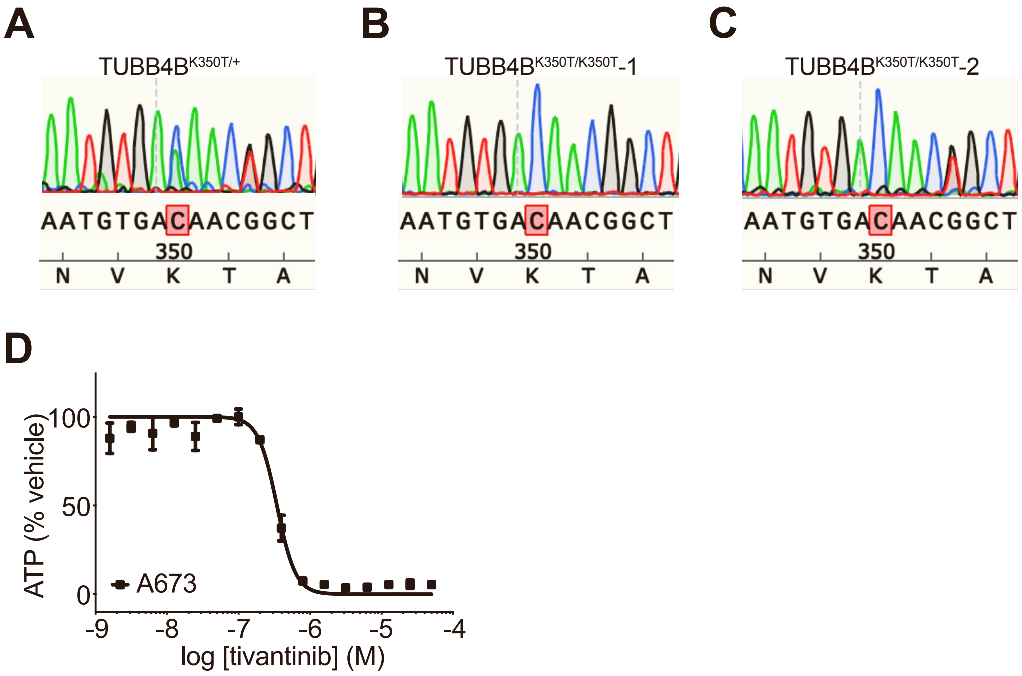

Figure S3, related to Figures 2 and 4. (A-C) Sequencing traces for *TUBB4B*^{K350T} mutation in three independent clones of EWS cells. (D) Dose-response curve for Tivantinib against EWS A673 cells.

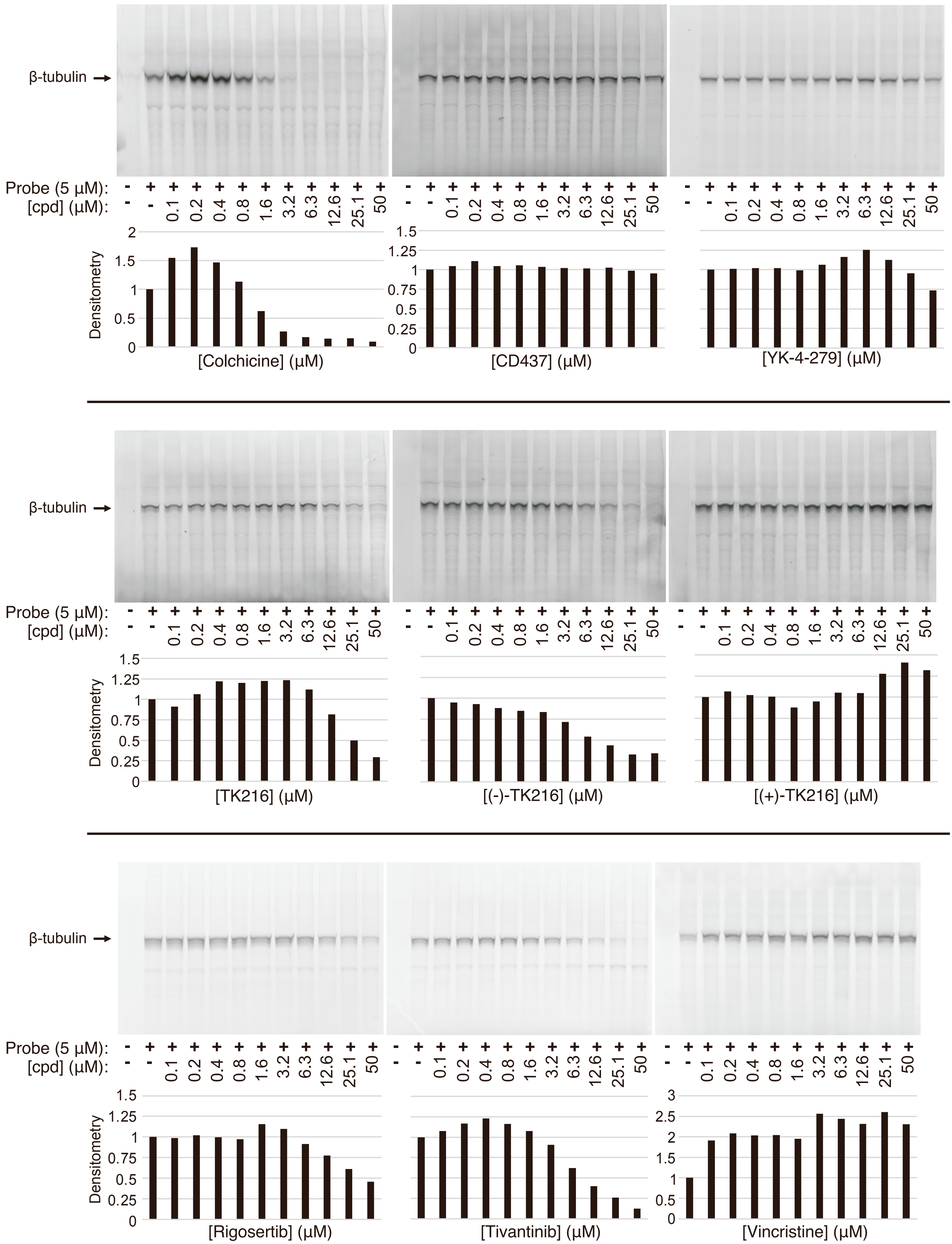

Figure S4, related to Figure 4. Full gel images of cell-based competition assay using covalent β-tubulin probe to assess tubulin binding in EWS cells by compounds, colchicine, CD437, YK-4-279, TK216, (-)-TK216, (+)-TK216, rigosertib, tivantinib, and vincristine. Densitometry graphs under gels were generated using ImageJ to measure intensity of β-tubulin band. Densitometry data were normalized to the control no competitor plus probe lane.

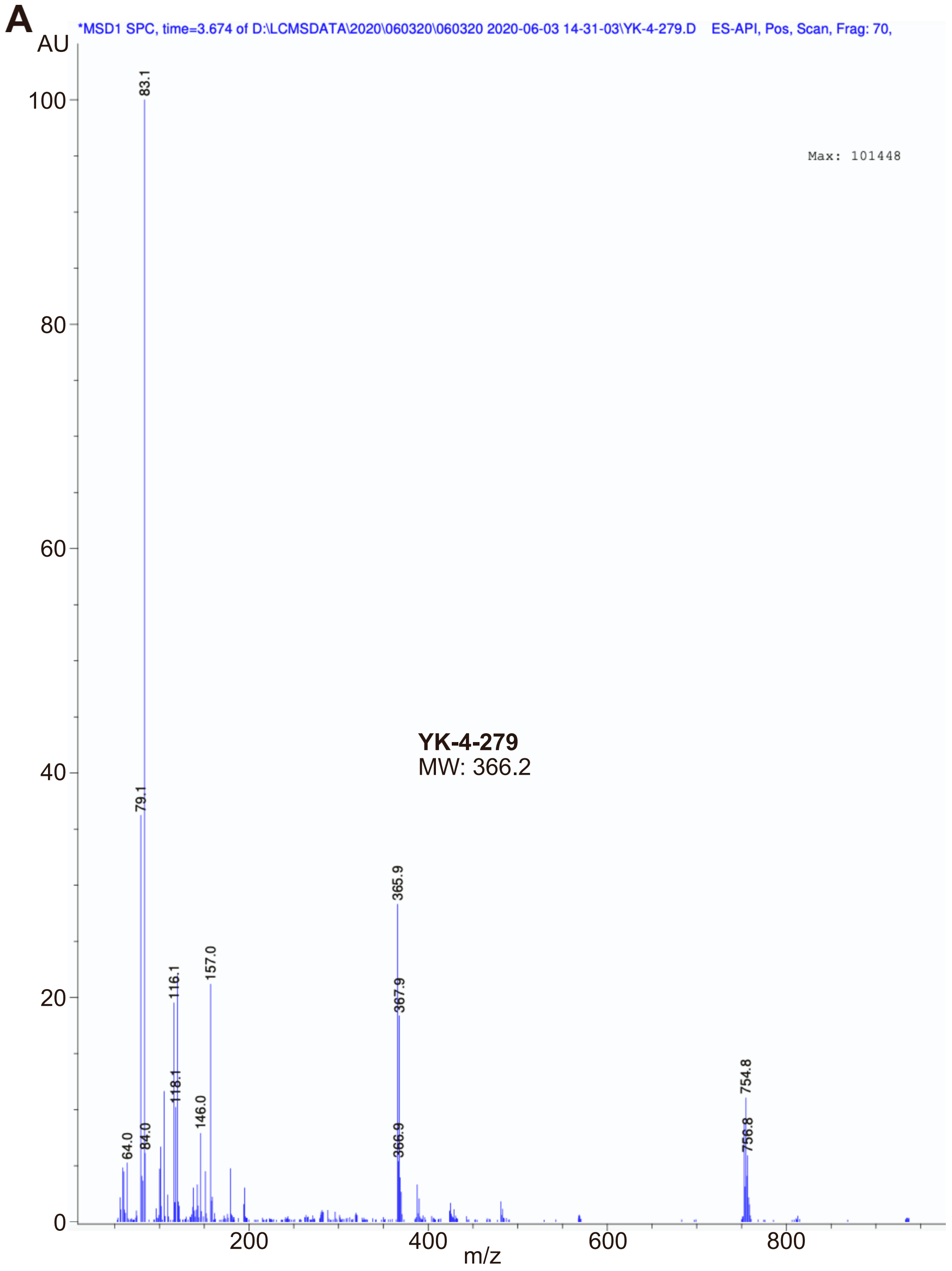

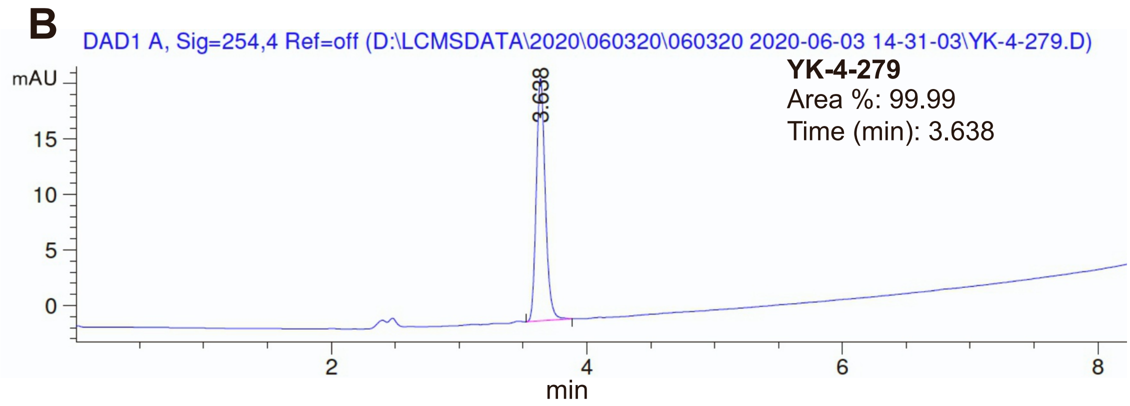

Data S1. HPLC (A) and HRMS (B) data of YK-4-279. Related to Figures 2, 3, and 4

A

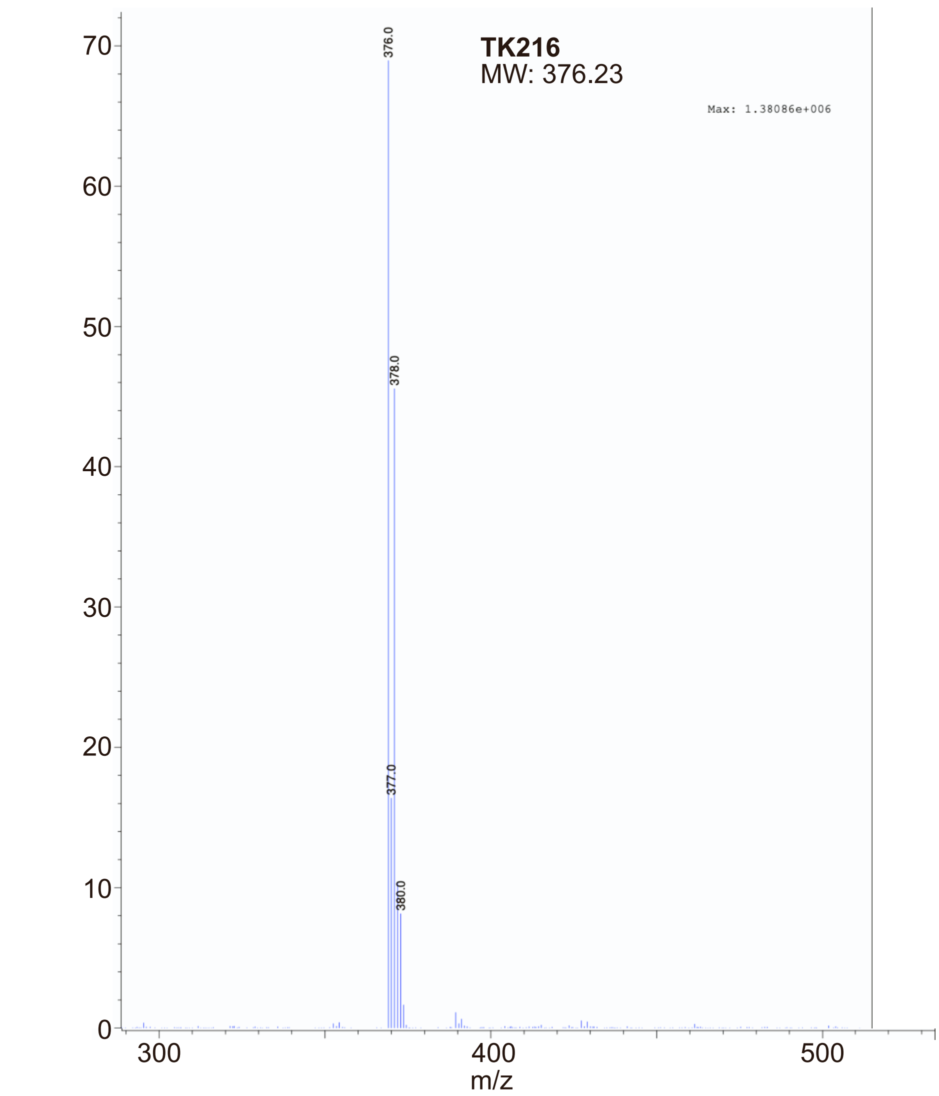

B

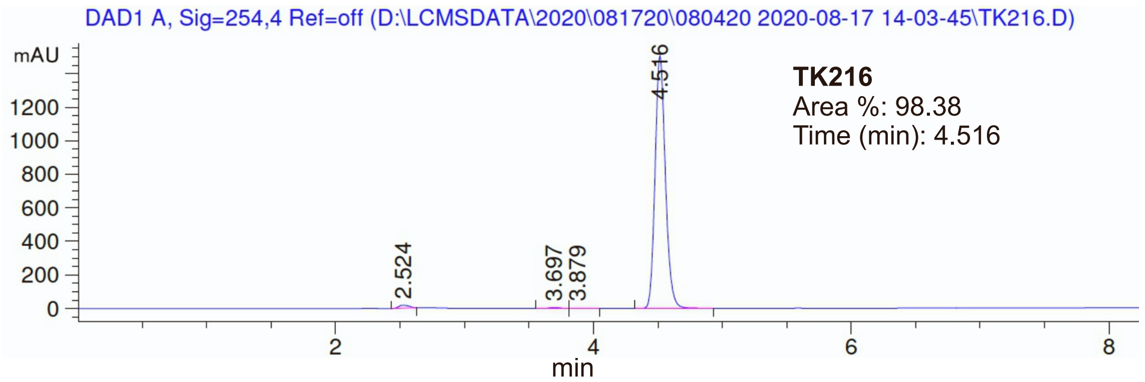

C

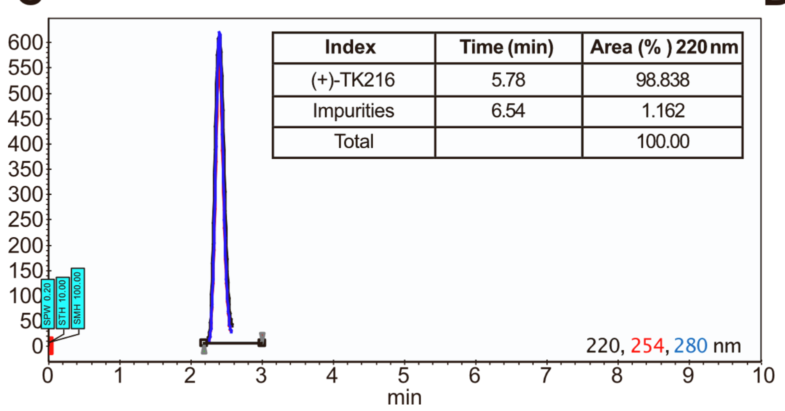

Index	Time (min)	Area (%) 220 nm
(+)-TK216	5.78	98.838
Impurities	6.54	1.162
Total		100.00

D

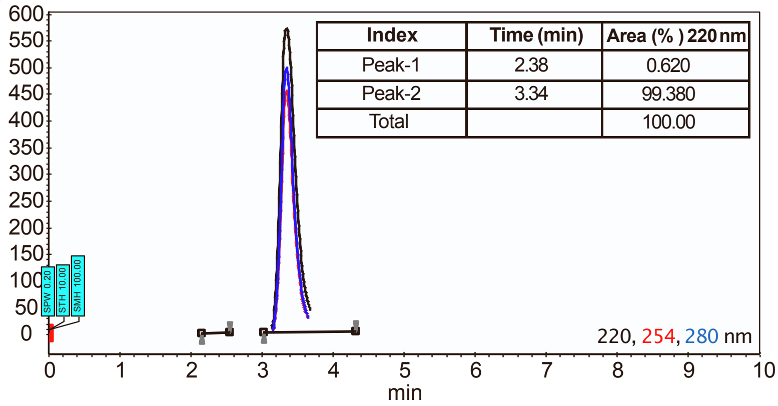

Index	Time (min)	Area (%) 220 nm
Peak-1	2.38	0.620
Peak-2	3.34	99.380
Total		100.00

Data S2. HRMS (A) and HPLC (B) data of TK216, and HPLC data for (+)-TK216 (C) and (-)-TK216 (D). Related to Figures 1, 2, 3, and 4.
