# Supplementary material for: TK216 targets microtubules in Ewing sarcoma cells
Source: Cell Chem Biol. 2022 Aug 18;29(8):1325–1332.e4. doi: 10.1016/j.chembiol.2022.06.002 (PMC9394687; doi:10.1016/j.chembiol.2022.06.002)
Supplement: Document S2. Article plus supplemental information [file mmc4.pdf]

Brief Communication

Cell Chemical Biology

TK216 targets microtubules in Ewing sarcoma cells

Graphical abstract

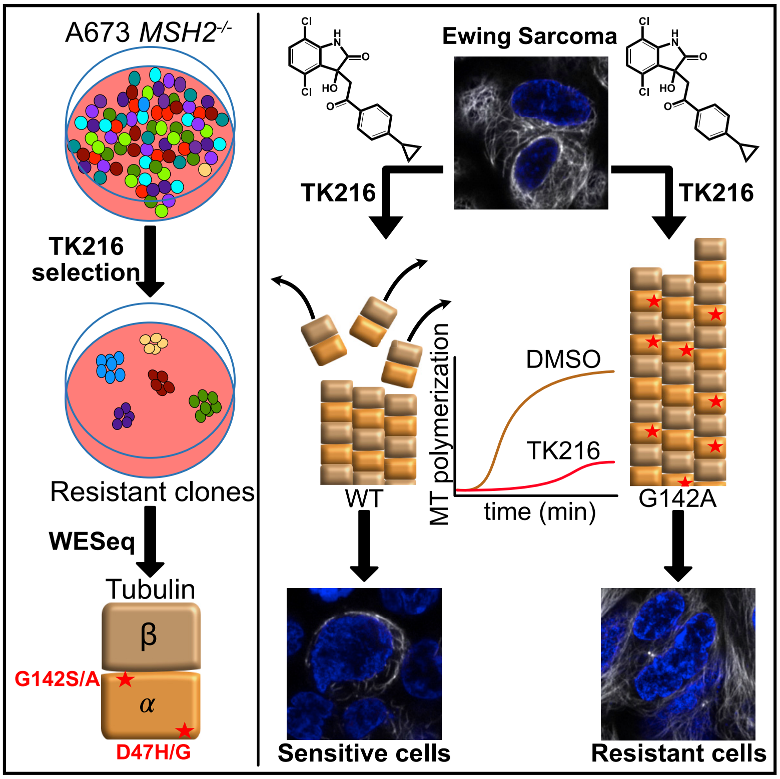

Authors

Juan Manuel Povedano, Vicky Li, Katherine E. Lake, ..., Yang Xie, Jef K. De Brabander, David G. McFadden

Correspondence

david.mcfadden@utsouthwestern.edu

In brief

TK216 (ONCT-216) was developed to inhibit the fusion protein that drives Ewing sarcoma, EWSR1-FLI1. However, the mechanism underlying TK216-induced cytotoxicity remained elusive. Here, Povedano et al. demonstrate using forward genetics, biochemical reconstitution, and chemical probe competition that TK216 acts as a microtubule destabilizing agent.

Highlights

- Recurrent *TUBA1B* mutations were identified in TK216-resistant Ewing sarcoma cells
- *TUBA1B* mutations independently drove resistance to TK216
- TK216 suppressed tubulin polymerization *in vitro* in a stereo-selective manner
- TK216 and vincristine act on microtubules through distinct binding mechanisms

Povedano et al., 2022, Cell Chemical Biology *29*, 1325–1332
August 18, 2022 © 2022 The Authors. Published by Elsevier Ltd.
https://doi.org/10.1016/j.chembiol.2022.06.002

CellPress

Cell Chemical Biology

CellPress
OPEN ACCESS

Brief Communication

TK216 targets microtubules in Ewing sarcoma cells

Juan Manuel Povedano,[1,2] Vicky Li,[1,2] Katherine E. Lake,[1,2] Xin Bai,[1,2] Rameshu Rallabandi,[2,3,4] Jiwoong Kim,[5] Yang Xie,[5] Jef K. De Brabander,[2,3,4] and David G. McFadden[1,2,3,4,6,*]
[1]Department of Internal Medicine, Division of Endocrinology, University of Texas Southwestern Medical Center, Dallas, TX 75390 USA
[2]Department of Biochemistry, University of Texas Southwestern Medical Center, Dallas, TX 75390 USA
[3]Harold C. Simmons Comprehensive Cancer Center, University of Texas Southwestern Medical Center, Dallas, TX 75390 USA
[4]Program in Molecular Medicine, University of Texas Southwestern Medical Center, Dallas, TX 75390 USA
[5]Department of Population and Data Sciences, University of Texas Southwestern Medical Center, Dallas, TX 75390 USA
[6]Lead contact
*Correspondence: david.mcfadden@utsouthwestern.edu
https://doi.org/10.1016/j.chembiol.2022.06.002

SUMMARY

Ewing sarcoma (EWS) is a pediatric malignancy driven by the EWSR1-FLI1 fusion protein formed by the chromosomal translocation t(11; 22). The small molecule TK216 was developed as a first-in-class direct EWSR1-FLI1 inhibitor and is in phase II clinical trials in combination with vincristine for patients with EWS. However, TK216 exhibits anti-cancer activity against cancer cell lines and xenografts that do not express EWSR1-FLI1, and the mechanism underlying cytotoxicity remains unresolved. We apply a forward-genetics screening platform utilizing engineered hypermutation in EWS cell lines and identify recurrent mutations in *TUBA1B*, encoding α-tubulin, that prove sufficient to drive resistance to TK216. Using reconstituted microtubule (MT) polymerization *in vitro* and cell-based chemical probe competition assays, we demonstrate that TK216 acts as an MT destabilizing agent. This work defines the mechanism of cytotoxicity of TK216, explains the synergy observed with vincristine, and calls for a reexamination of ongoing clinical trials with TK216.

INTRODUCTION

Ewing sarcoma (EWS) is defined by chromosomal translocations that lead to the expression of oncogenic fusion proteins of the EWSR1-FLI1 family. The EWSR1-FLI1 family of proteins is formed by the fusion of protein sequences of low complexity from EWSR1, FUS, or TAF15 to the DNA-binding domain of an E26-transformation-specific (ETS) family transcription factor, most frequently FLI1 or ERG (Riggi et al., 2021). The EWSR1-FLI1 protein lacks known enzymatic activity or defined small-molecule binding pockets; therefore, rational therapeutic targeting of the protein represents a major challenge.

YK-4-279 is a small molecule reported to directly target EWSR1-FLI1. It was identified as a small molecule capable of disrupting an interaction between EWSR1-FLI1 and RNA helicase A (encoded by *DHX9*). YK-4-279 induced apoptotic cell death in EWS cell lines and suppressed growth of EWS xenografts (Erkizan et al., 2009). YK-4-279 was subsequently shown to induce G_2-M cell-cycle arrest and apoptosis in synergy with the microtubule (MT) destabilizing agent vincristine (Zollner et al., 2017). TK216 (also called ONCT-216), a clinical derivative of YK-4-279, has entered phase II clinical trials in patients with EWS as monotherapy and in combination with vincristine, and a subset of patients have exhibited promising responses (Ludwig et al., 2021).

Since the original publication of YK-4-279 as an inhibitor of EWSR1-FLI1, the molecule has been shown to suppress growth of a variety of cancer cell lines not driven by EWSR1-FLI1, including prostate cancer, neuroblastoma, lymphoma, melanoma, and thyroid cancer (Huang et al., 2021; Kollareddy et al., 2017; Rahim et al., 2014; Spriano et al., 2019; Xue et al., 2021). In addition, genetic suppression of EWSR1-FLI1 induced cell-cycle arrest at a different checkpoint: the G_1-S transition (Hu et al., 2008). These findings have raised questions regarding the mechanism underlying cytotoxicity induced by YK-4-279. Considering the ongoing clinical trials using TK216, an urgent need exists to define the direct mechanism by which this drug induces cytotoxicity.

RESULTS

We previously reported that engineered DNA mismatch repair (MMR) deficiency induces hypermutation in cancer cell lines that facilitates the emergence of compound-resistant alleles (Povedano et al., 2019). These mutations can reveal the direct protein targets of cytotoxic small molecules (Han et al., 2016, 2017; Lin et al., 2019a; Povedano et al., 2019, 2020; Wacker et al., 2012). We sought to uncover the mechanism of TK216-induced cytotoxicity using this unbiased forward-genetics platform. We performed forward-genetic screening with TK216 using MMR-deficient A673 EWS cells, A673-M1, at three concentrations

© 2022 The Authors. Published by Elsevier Ltd.
This is an open access article under the CC BY license (http://creativecommons.org/licenses/by/4.0/).

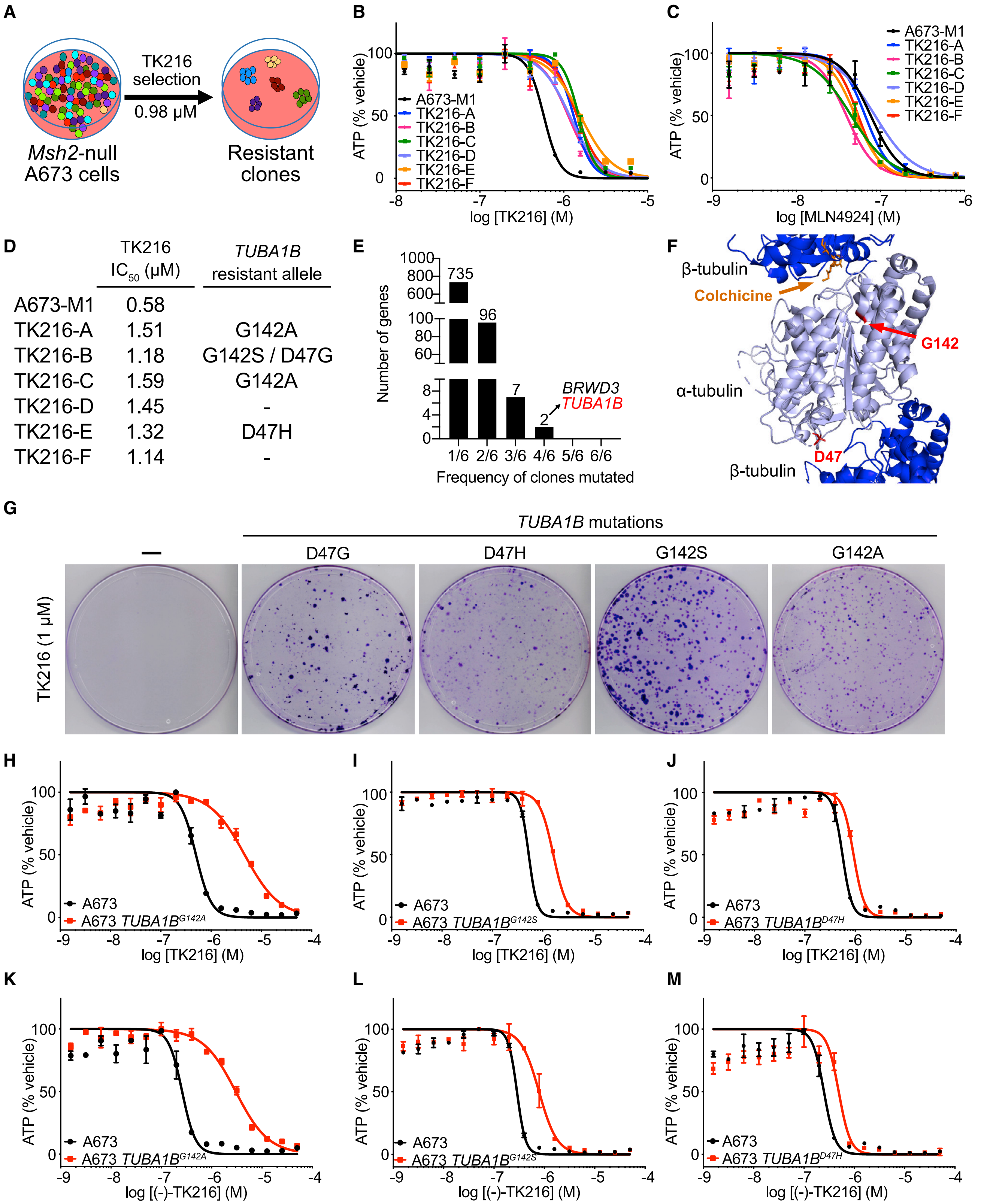

Figure 1. Forward-genetic screening for TK216 resistance identifies *TUBA1B* mutations sufficient to confer resistance to YK-4-279/TK216

(A) Workflow for forward-genetic screening using *Msh2*-null EWS cells, A673-M1.

(B and C) Dose-response curves for TK216 (B) and MLN4924 (C) against parental A673-M1 and compound-resistant clones.

(legend continued on next page)

CellPress
OPEN ACCESS

flanking the IC_{100}^{1wk}, the minimum concentration at which no viable cells were observed after 1 week of compound exposure (Figure 1A; STAR Methods). Six compound-resistant clones (TK216 A–F) emerged following TK216 selections. We confirmed resistance to TK216 in all six clones (range 1.98- to 2.74-fold) (Figures 1B and 1D). To ensure that generalized mechanisms of resistance did not underlie the emergence of TK216-resistant clones, we tested the unrelated anti-cancer toxins etoposide and MLN4924 and confirmed that the clones were specifically resistant to TK216 (Figures 1C and S1A).

We identified recurrently mutated genes in the TK216-resistant clones by exome sequencing (Han and Nijhawan, 2019). Two genes, *TUBA1B* and *BRWD3*, were mutated in four of six clones, with no other gene recurrently mutated in more than four clones (Figure 1E; Data S1). No mutations were identified in *EWSR1*, *FLI1*, or *DHX9* (Table S1; Data S1). We compared somatic mutations between TK216-resistant clones to establish whether clones were related or arose independently. TK216-A and TK216-C clones shared 91 mutations, suggesting that these clones were closely related. No other clones shared more than 11 somatic mutations. We prioritized *TUBA1B*, encoding α-tubulin, as a candidate because independent clones harbored recurrent mutations in two codons leading to different amino acid substitutions, G142 A/S and D47 G/H. The observation of different substitutions of the same codon suggested strong selective pressure for alteration of these specific residues in the presence of TK216 (Figures 1D–1F). The G142S mutation was also previously reported to confer resistance to the MT destabilizing agent dinitroaniline, raising the possibility that TK216 targeted MTs (Ma et al., 2008).

We used CRISPR-Cas9 to engineer each mutation into drug-naïve, MMR-intact, A673 EWS cells to determine whether *TUBA1B* codon 47 and 142 mutations were sufficient to induce resistance to YK-4-279 and TK216 (STAR Methods). Cells nucleofected with CRISPR-Cas9 components were selected with TK216 (1 μM) for 2 weeks followed by crystal violet staining. Emerging clones were observed in cells transfected with the *TUBA1B* mutation repair templates, whereas no clones were visible in the control condition without Cas9 protein or the repair template (Figure 1G). This result suggested that *TUBA1B* codon 47 or 142 mutations were sufficient to confer resistance to TK216.

We expanded pools of engineered $TUBA1B^{G142}$ and $TUBA1B^{D47}$ cells and validated mutations by Sanger sequencing (Figures S1B–S1D). The $TUBA1B^{G142A}$ mutation appeared homozygous, whereas the $TUBA1B^{G142S}$ and $TUBA1B^{D47H}$ mutations were heterozygous or present in one-third of alleles, respectively. $TUBA1B^{G142S}$, $TUBA1B^{G142A}$, and $TUBA1B^{D47H}$ mutations were independently sufficient to confer resistance to YK-4-279 and TK216 (Figures 1H–1J and S1E–S1G). $TUBA1B^{G142A}$ cells exhibited the greatest degree of resistance to TK216, possibly relating to homozygosity of the engineered mutation. A673-$TUBA1B^{G142A}$, -$TUBA1B^{G142A}$, and -$TUBA1B^{D47H}$ cells were not resistant to the DNA polymerase α inhibitor CD437 or the neddylation-activating enzyme inhibitor MLN4924 (Figures S1H–S1M).

Both YK-4-279 and TK216 contain a chiral center, and previous studies demonstrated that the (−)-YK-4-279 enantiomer was responsible for the anti-cancer activity in EWS cells (Barber-Rotenberg et al., 2012). We separated (+)- and (−)-TK216 enantiomers to 98.8% and 99.4% purity, respectively, using supercritical fluid chromatography (SFC) (Data S2). Consistent with prior reports, (−)-TK216 enantiomer exhibited 56-fold greater activity in EWS cells (half-maximal inhibitory concentrations [IC_{50}] = 0.26 μM) compared with the (+)-TK216 enantiomer (IC_{50} = 14.57 μM) (Figures S1N and S1O). $TUBA1B^{G142S}$, $TUBA1B^{G142A}$, and $TUBA1B^{D47H}$ mutations were also sufficient to confer resistance to purified (−)-TK216 enantiomer (Figures 1K–1M). Thus, introduction of a single *TUBA1B* mutation identified by forward-genetics screening in EWS cells was sufficient to endow resistance to YK-4-279, TK216, and (−)-TK216 enantiomer.

Review of the crystal structure of the α-tubulin:β-tubulin dimer placed G142 and D47 at opposite interfaces of the heterodimer, near the GTP pocket of α-tubulin and the interface of the α- and β-tubulin heterodimers, respectively. Both amino acids were outside previously described binding sites for MT targeting agents (Figures S2A–S2C). Together, these findings made it unlikely that these mutations impaired interaction of TK216 by altering a single binding pocket (Figure 1F).

We hypothesized that TK216 might act as an MT destabilizing agent and that G142 and D47 mutations induced resistance to TK216 by stabilizing MTs. We therefore tested whether G142A, G142S, and D47H mutations also conferred resistance to colchicine, a potent natural product MT destabilizer through which the eponymous binding pocket was defined. Indeed, A673-$TUBA1B^{G142A}$, -$TUBA1B^{G142A}$, and -$TUBA1B^{D47H}$ cells exhibited resistance to colchicine (Figures 2A–2C). We also tested whether the G142A mutation was capable of driving resistance to vincristine, which destabilizes MTs through a binding pocket distinct from colchicine. A673-$TUBA1B^{G142A}$ cells exhibited resistance to vincristine (IC_{50} = 1.8 nM) compared with A673 cells (IC_{50} = 0.5 nM) (Figure S2D).

Mutations in lysine 350 of β-tubulin stabilize MTs and endow resistance to destabilizing agents including colchicine (Hua et al., 2001; Schibler and Huang, 1991). To further assess if YK-4-279 and TK216 acted as MT destabilizing agents, we engineered the K350T mutation into *TUBB4B*, the highest expressed isoform of β-tubulin in drug-naïve A673 EWS cells (data not shown). We obtained one heterozygous and two homozygous $TUBB4B^{K350T}$ cell lines

(D) Table with half-maximal inhibitory concentrations (IC_{50}) for TK216 against parental A673-M1 and compound-resistant clones.
(E) *TUBA1B* is one of two genes recurrently mutated in four out of six clones.
(F) Co-crystal structure of α-/β-tubulin with colchicine (PDB: 1Z2B). G142 and D47 are shown in red, and colchicine is depicted in orange.
(G) Crystal violet staining of EWS cells harboring *TUBA1B* D47 G/H or G142 S/A mutations following 2 weeks of TK216 selection.
(H–J) Dose-response curves for TK216 against EWS cells harboring $TUBA1B^{G142A}$, $TUBA1B^{G142S}$, and $TUBA1B^{D47H}$ mutations.
(K–M) Dose-response curves for (−)-TK216 against EWS cells harboring $TUBA1B^{G142A}$, $TUBA1B^{G142S}$, and $TUBA1B^{D47H}$ mutations.
Dose-response curves were performed at least twice for each compound and in duplicate per concentration. Data are presented as mean ± SD. One representative experiment is shown in each figure.
See also Figures S1 and S2.

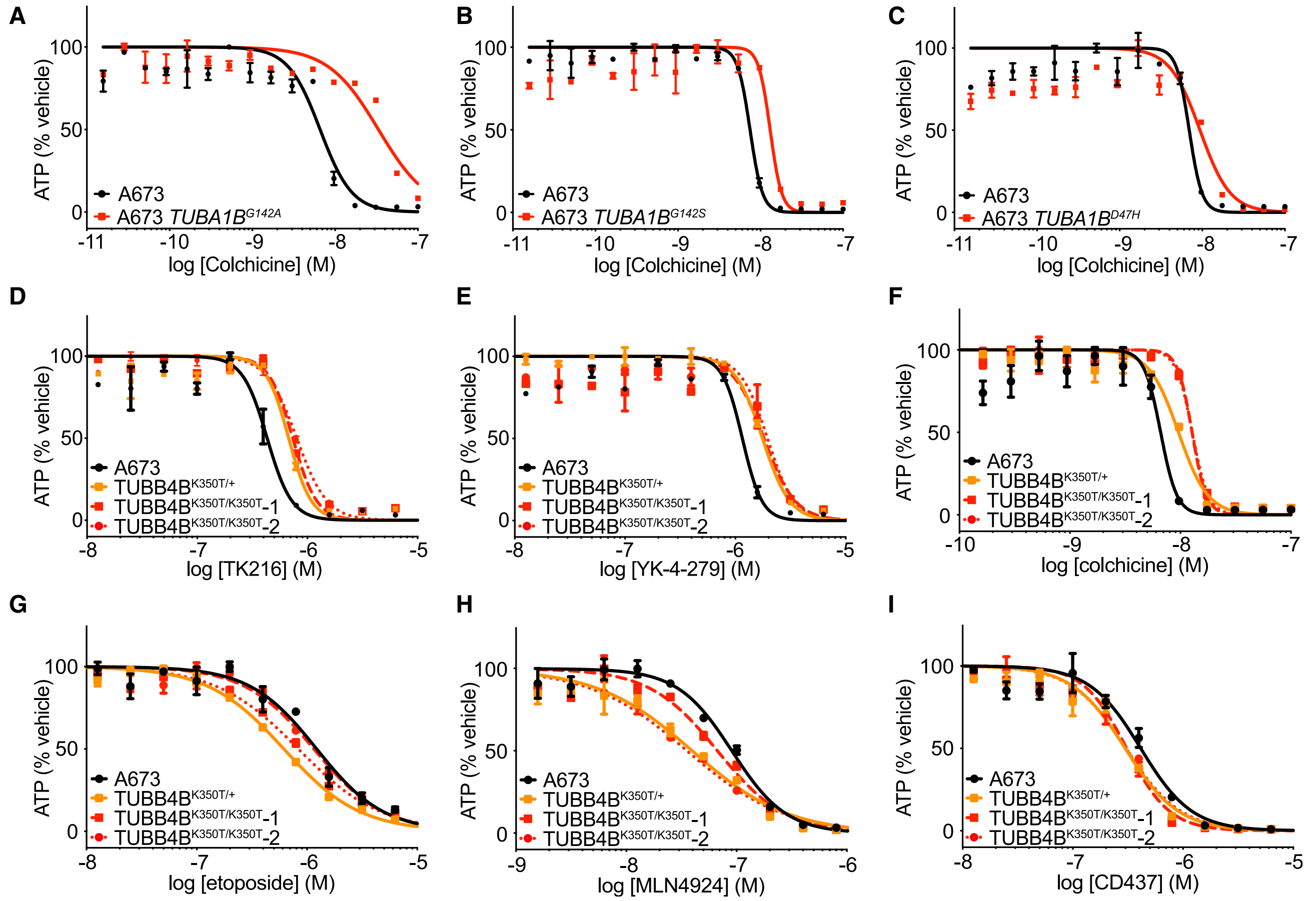

Figure 2. Mutations in α- and β-tubulin confer resistance to YK-4-279 and TK216

(A–C) Dose-response curves for colchicine against A673 cells harboring *TUBA1B*^{G142A}, *TUBA1B*^{G142S}, and *TUBA1B*^{D47H} mutations.

(D–I) Dose-response curves for TK216, YK-4-279, colchicine, etoposide, MLN4924, and CD437 against *TUBB4B*$^{K350T/+}$, *TUBB4B*$^{K350T/K350T}$*-1*, and *TUBB4B*$^{K350T/K350T}$*-2* EWS cells.

Each experiment was performed at least twice and in duplicate per concentration. Data are presented as mean ± SD. One representative experiment is shown. See also Figure S3.

(Figures S3A–S3C). Both K350T/+ and K350T/K350T mutations in *TUBB4B* conferred resistance to YK-4-279, TK216, and colchicine (Figures 2D–2F). In contrast, K350T mutations did not confer resistance to cytotoxins acting independently of MTs including etoposide (topoisomerase II inhibitor), MLN4924 (NEDD8 activating enzyme inhibitor), or CD437 (POLA1 inhibitor) (Figures 2G–2I).

These studies suggested a model in which the anti-proliferative activity of TK216 stemmed from action as an MT destabilizing agent. We sought to reconstitute MT polymerization *in vitro* to directly assess whether YK-4-279 and TK216 altered MT function. MTs are dynamic structures composed of α-tubulin:β-tubulin heterodimers that polymerize and de-polymerize through a phenomenon called dynamic instability (Brouhard and Rice, 2018). We used an MT turbidity assay to determine whether YK-4-279 and TK216 impacted MT dynamics. This assay measures the formation of MT polymers by reading absorbance of a mixture of α-tubulin:β-tubulin heterodimers in conditions that facilitate polymerization. Using a molar ratio of compound:tubulin of 1:5 (25 μM of cycled tubulin), 5 μM of YK-4-279 and TK216 delayed MT polymerization. The positive control, colchicine (5 μM), also delayed MT polymerization (Figure 3A). Inhibition of MT polymerization was evident with 0.5 μM of TK216 and increased in a dose-dependent manner (Figure 3B). To exclude nonspecific assay interference, we tested CD437 (5 μM) and observed no impact on MT polymerization (Figure 3A).

We next evaluated whether inhibition of MT polymerization was enantiomer specific. Indeed, (–)-TK216 potently inhibited MT polymerization whereas (+)-TK216 did not (Figure 3C). Therefore, the anti-proliferative activity and inhibition of MT polymerization were both unique properties of the (–)-enantiomer of TK216. We also visualized the impact of (–)-TK216 and colchicine on A673 and A673-*TUBA1B*^{G142A} cells. Following incubation with compound for 20 h, we stained the cells with Tubulin Tracker Deep Red and examined cells using super-resolution microscopy. We observed MT disruption in A673 cells treated with (–)-TK216 and colchicine compared with DMSO control. In addition, MTs from A673-*TUBA1B*^{G142A} cells were longer and more organized

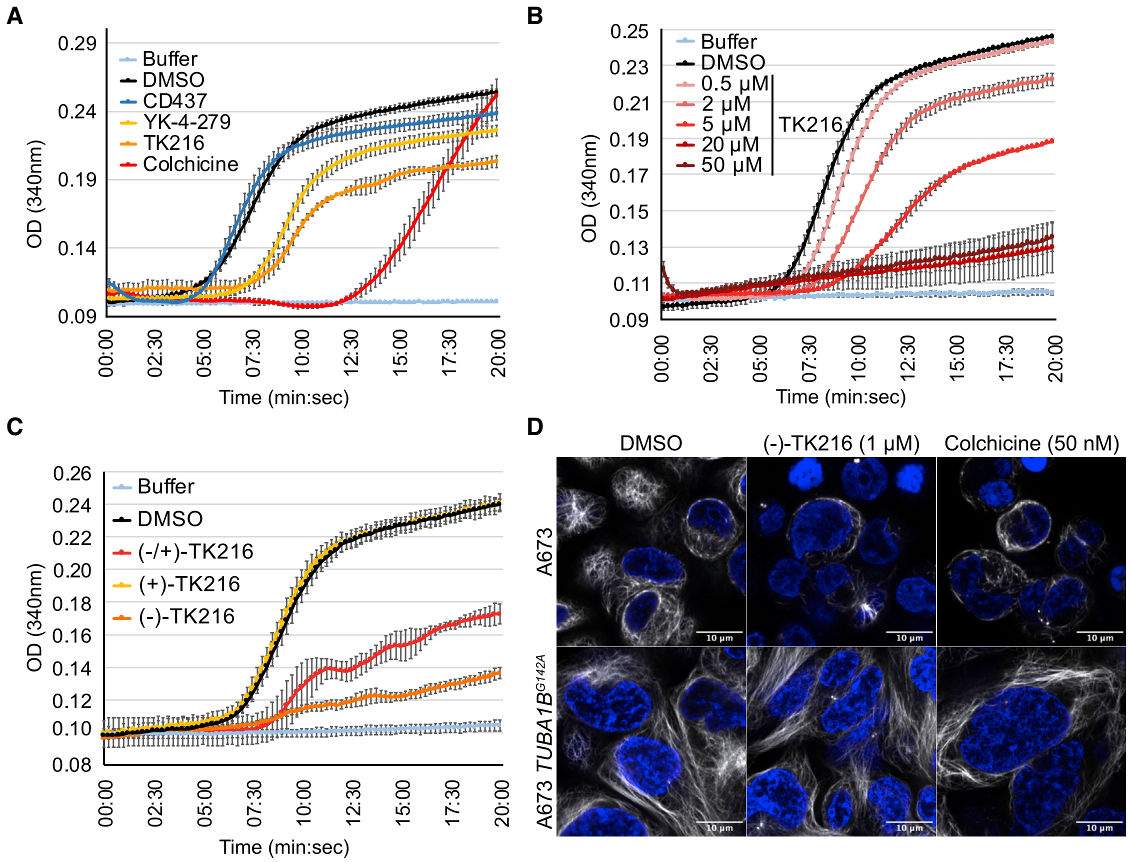

Figure 3. TK216 suppresses MT polymerization *in vitro* and alters MT structure in cells

(A) MT polymerization assay following addition of DMSO, CD437, YK-4-279, TK216, or colchicine at 5 µM.

(B) Dose-dependent inhibition of MT polymerization by TK216 treatment.

(C) Inhibition of MT polymerization by (−)-TK216 enantiomer compared to inactive (+)-TK216 enantiomer.

(D) Super-resolution microscopy of microtubules (white) stained with Tubulin Tracker Deep Red and nuclei (blue) from A673 and A673-*TUBA1B*^{G142A} cells treated for 20 h with DMSO, (−)-TK216, and colchicine.

Each experiment was performed three times. Data are presented as mean ± SD. One representative experiment is shown in each figure.

compared with wild-type A673 cells (Figure 3D). Following administration of TK216 or colchicine, we observed maintenance of MTs in A673-*TUBA1B*^{G142A} cells compared with wild-type A673 cells. These data supported the hypothesis that *TUBA1B*^{G142A} mutation counteracts the effect of (−)-TK216 and colchicine through stabilization of MTs.

Treatment of cells and xenografts with YK-4-279 was reported to synergize with vincristine; however, the mechanism underlying synergy has not been elucidated. If YK-4-279 and vincristine both target MTs, how can synergy between these agents be explained? Distinct chemical families target MTs through several different binding pockets (Florian and Mitchison, 2016; Wordeman and Vicente, 2021). We hypothesized that synergy between TK216 and vincristine could be explained if these agents acted on distinct MT binding pockets. We previously reported the development of a tubulin chemical probe that covalently modifies Cys239 within the colchicine binding pocket of β-tubulin (Povedano et al., 2020). We developed a cell-based competition assay and showed that small molecules acting through the colchicine binding pocket competed the benzamide probe whereas vincristine, which acts through a separate vinca alkaloid binding pocket, did not (Povedano et al., 2020).

We tested colchicine binding pocket agents including colchicine, rigosertib, and tivantinib (Aoyama et al., 2014; Jost et al., 2017, 2020; Xiang et al., 2015) in the probe competition assay. Indeed, colchicine (IC_{50} for cytotoxicity, 7 nM), rigosertib (IC_{50} for cytotoxicity, 100 nM), and tivantinib (IC_{50} for cytotoxicity, 330 nM) competed the tubulin chemical probe at concentrations above 0.8, 6.3, and 3.2 µM, respectively. In contrast, vincristine did not compete the benzamide probe at concentrations up to 50 µM, which was well above the IC_{50} for cytotoxicity (1 nM) (Figures 4A, S3D, and S4). We also tested CD437, which exhibited no probe competition at concentrations up to 50 µM (IC_{50} for cytotoxicity, 600 nM) (Figures 4A and S4).

We next determined whether YK-4-279 and TK216 competed the tubulin chemical probe. Both compounds competed the benzamide probe at concentrations above 12.5 (YK-4-279) and 6.3 µM (TK216) (Figures 4B and S4). We also performed competition assays using TK216 enantiomers (Figure 4B). (−)-TK216 competed the benzamide probe at concentrations above

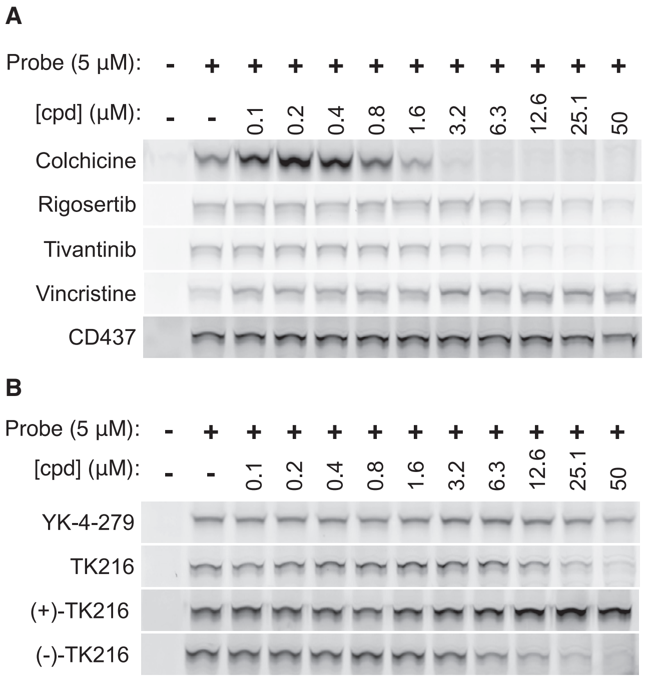

Figure 4. TK216 binds MTs through a distinct mechanism from vincristine

(A) Cell-based probe competition assay of tubulin binding by colchicine, rigosertib, tivantinib, vincristine, and CD437.

(B) Cell-based probe competition assays using YK-4-279, TK216, (+)-TK216, and (−)-TK216.

All experiments were performed at least twice. One representative experiment is shown in each figure.

See also Figure S4.

1.6 μM, whereas (+)-TK216 was devoid of competition activity at concentrations up to 50 μM (Figures 4B and S4). Therefore, YK-4-279/TK216 and vincristine destabilize MTs through distinct binding mechanisms.

DISCUSSION

Each experimental approach presented here, including unbiased forward genetics, reconstituted MT polymerization, and cell-based chemical probe assays converged upon the conclusion that TK216 exhibits anti-proliferative activity by acting as an MT destabilizing agent. Can these findings be reconciled with the scientific literature suggesting that YK-4-279 and TK216 act directly on the EWSR1-FLI1 fusion protein? YK-4-279 was identified using a surface plasmon resonance assay to identify small molecules capable of binding recombinant EWSR1-FLI1 protein (Erkizan et al., 2009). At high concentrations *in vitro* (30 μM), YK-4-279 was found to displace binding of a 10 amino acid peptide from RNA helicase A to recombinant EWSR1-FLI1. The authors also reported that YK-4-279 blocked immunoprecipitation of RNA helicase A and EWSR1-FLI1 in EWS cells and suppressed EWSR1-FLI1-dependent transcription of the *NR0B1* promoter, a validated EWSR1-FLI1 transcriptional target (Selvanathan et al., 2015).

Treatment of EWS cells with YK-4-279 induced cell-cycle arrest at the G_2-M transition, a hallmark phenotype of MT agents. In contrast, genetic suppression of EWSR1-FLI1 induced cell-cycle arrest at a different checkpoint: the G_1-S transition (Hu et al., 2008). In addition, after the publication of Erkizan et al., a series of publications from independent laboratories demonstrated that YK-4-279 induced cell death in several different cancer types, including prostate cancer, neuroblastoma, lymphoma, melanoma, and thyroid cancer (Huang et al., 2021; Kollareddy et al., 2017; Rahim et al., 2014; Spriano et al., 2019; Xue et al., 2021). The discordance in the phenotypes induced by YK-4-279 and genetic suppression of EWSR1-FLI1, the induction of G_2-M cell-cycle arrest, and the broad anti-proliferative activity of YK-4-279 are consistent with the data presented here and the conclusion that YK-4-279 acts as an MT destabilizing agent. Nevertheless, we do not exclude the possibility of TK216 also binds and acts on EWSR1-FLI1 and/or DHX9. However, additional medicinal chemistry efforts will be required to decouple such activity from MT destabilization.

Based on our chemical probe competition assays, we favor the hypothesis that TK216 acts on tubulin through the colchicine binding site. However, the possibility remains that TK216 binds outside the colchicine pocket and displaces the benzamide probe through an allosteric, or indirect, mechanism. It was of interest that we identified recurrent mutations impacting α-tubulin, rather than β-tubulin, in TK216-resistant A673 clones. The colchicine binding pocket is encoded within β-tubulin, not α-tubulin (McLoughlin and O'Boyle, 2020). If TK216 competes our colchicine binding pocket chemical probe, then why were mutations in β-tubulin not identified? β- and α-tubulins are each expressed as multiple isoforms from eight genes. Expression of different tubulin genes varies between cell lineages and cancer cell lines, and many cell lines express relatively uniform levels of multiple β- and α-tubulin genes. Therefore, a compound-resistant allele in a single tubulin gene is expected to result in limited impact on MT dynamics or resistance to an MT agent. We reviewed β- and α-tubulin isoform expression from a previously reported study of A673 cells (Lin et al., 2019b; Data S1). We found that β-tubulin isoforms were expressed at similar levels in A673 cells, whereas *TUBA1B* was the dominantly expressed α-tubulin. We hypothesize that the emergence of *TUBA1B* mutations, rather than β-tubulin gene mutations, stems from greater impact of a single mutation in *TUBA1B* on the cellular pool of MTs.

This study raises the possibility that agents targeting MTs through the colchicine pocket might offer clinical benefit in EWS and other tumors. Treatment with TK216 and vincristine induced tumor regression in a subset of patients enrolled in the phase I/II trial (Ludwig et al., 2021). Our observation that TK216 and vincristine act through different binding sites provides a mechanistic explanation for the clinical activity of this combination. Rigosertib, which was initially developed as a Polo-like kinase 1 (PLK1) inhibitor, was subsequently shown to act as an MT destabilizing agent through the colchicine binding pocket (Aoyama et al., 2014; Jost et al., 2017, 2020; Xiang et al., 2015). Rigosertib, which has advanced to phase III clinical trials, likely represents the first colchicine binding pocket agent to exhibit a reasonable safety profile. The experience with rigosertib, and now TK216, highlights the importance of carefully uncovering the cellular mechanism of action of small molecule

therapeutics developed from target-based *in vitro* screening. However, the activity of these compounds in clinical trials also hints that MT agents acting through the colchicine binding pocket might offer clinical benefit in EWS and other cancers, alone or in combination with MT agents acting through separate binding pockets such as vincristine.

Limitations of the study

This study establishes that TK216 acts as an MT destabilizing agent in EWS cells and that this activity underlies cytotoxicity. However, the work does not exclude the possibility that TK216 also binds and acts on EWSR1-FLI1 and/or DHX9. Additional medicinal chemistry efforts will be required to decouple such activity from MT destabilization. This work also uses largely indirect measures for TK216 binding through the colchicine binding site (chemical probe competition), and structural studies would be required to resolve the exact mechanism by which TK216 destabilizes MTs.

SIGNIFICANCE

TK216 is a small molecule developed to directly target the EWSR1-FLI1 fusion oncoprotein that is the central genetic driver of EWS, and TK216 is currently in phase II clinical trials. However, TK216 exhibits toxicity to numerous cancer cell lines, including cell lines not driven by EWSR1-FLI1 or other ETS-family transcription factors. This has raised the concern that targeting of other cellular proteins by TK216 might account for the anti-proliferative activity of the molecule. We employ a combination of forward-genetics screening and cell-based chemical probe competition assays to determine the mechanism by which TK216 impairs cancer cell growth. These orthogonal approaches both converged on MTs as central to the mechanism of action of TK216. We reconstitute MT polymerization *in vitro* and demonstrate that TK216 acts as a MT destabilizing agent. Using cell-based chemical probe competition assays, we also uncover that TK216 binds to MTs through a distinct binding site compared with vincristine, a finding that might explain the synergy observed between these two molecules. These results call for a reexamination of ongoing clinical trials using TK216 and additional investigation of potential synergy between colchicine binding pocket agents and vinca alkaloids.

STAR★METHODS

Detailed methods are provided in the online version of this paper and include the following:

- KEY RESOURCES TABLE
- RESOURCE AVAILABILITY
  - Lead contact
  - Material availability
  - Data and code availability
- EXPERIMENTAL MODEL AND SUBJECT DETAILS
- METHOD DETAILS
  - Compounds
  - Forward genetic screen of TK216
  - Whole exome sequencing analysis
  - Introduction of tubulin mutations
  - Cytotoxic assay
  - MT polymerization assay
  - Cell-based tubulin probe competition assay
  - Microscopy of microtubules
- QUANTIFICATION AND STATISTICAL ANALYSIS

SUPPLEMENTAL INFORMATION

Supplemental information can be found online at https://doi.org/10.1016/j.chembiol.2022.06.002.

ACKNOWLEDGMENTS

This work was supported by grants from the Welch Foundation (I-2040, D.G.M. and I-1422, J.K.D.B.); the National Cancer Institute of the NIH (U54CA231649, D.G.M.); a Disease-Oriented Scholar Award from UT Southwestern (D.G.M.); a Clinical Investigator Award from the Damon Runyon Cancer Research Foundation (102-19, D.G.M.); the Cancer Prevention and Research Institute of Texas (RP190414, D.G.M.); and a Pilot Synergy Award from the UT Southwestern Dean's Circle of Friends (D.G.M. and J.K.D.B.). J.K.D.B. holds the Julie and Louis Beecherl, Jr., Chair in Medical Science. We thank Deepak Nijhawan for critically reading the manuscript and innumerable scientific discussions. We thank Katherine Luby-Phelps and the Quantitative Light Microscopy Core at UTSW for technical support (supported by NIH, 1S10OD021684-01).

AUTHOR CONTRIBUTIONS

D.G.M. conceived the study and supervised research. J.M.P. designed and performed experiments. K.E.L., V.L., and X.B. performed experiments. R.R. and J.K.D.B. designed and synthesized chemical probes. J.K. analyzed exome sequencing data. Y.X. supervised J.K. and exome sequencing analysis. D.G.M. and J.M.P. wrote the manuscript.

DECLARATION OF INTERESTS

The authors declare no competing interests.

Received: January 5, 2022
Revised: May 2, 2022
Accepted: June 15, 2022
Published: July 7, 2022

REFERENCES

Aoyama, A., Katayama, R., Oh-Hara, T., Sato, S., Okuno, Y., and Fujita, N. (2014). Tivantinib (ARQ 197) exhibits antitumor activity by directly interacting with tubulin and overcomes ABC transporter-mediated drug resistance. Mol. Cancer Ther. *13*, 2978–2990. https://doi.org/10.1158/1535-7163.MCT-14-0462.

Barber-Rotenberg, J.S., Selvanathan, S.P., Kong, Y., Erkizan, H.V., Snyder, T.M., Hong, S.P., Kobs, C.L., South, N.L., Summer, S., Monroe, P.J., et al. (2012). Single enantiomer of YK-4-279 demonstrates specificity in targeting the oncogene EWS-FLI1. Oncotarget *3*, 172–182. https://doi.org/10.18632/oncotarget.454.

Brouhard, G.J., and Rice, L.M. (2018). Microtubule dynamics: an interplay of biochemistry and mechanics. Nat. Rev. Mol. Cell Biol. *19*, 451–463. https://doi.org/10.1038/s41580-018-0009-y.

DePristo, M.A., Banks, E., Poplin, R., Garimella, K.V., Maguire, J.R., Hartl, C., Philippakis, A.A., del Angel, G., Rivas, M.A., Hanna, M., et al. (2011). A framework for variation discovery and genotyping using next-generation DNA sequencing data. Nat. Genet. *43*, 491–498. https://doi.org/10.1038/ng.806.

Erkizan, H.V., Kong, Y., Merchant, M., Schlottmann, S., Barber-Rotenberg, J.S., Yuan, L., Abaan, O.D., Chou, T.H., Dakshanamurthy, S., Brown, M.L., et al. (2009). A small molecule blocking oncogenic protein EWS-FLI1

interaction with RNA helicase A inhibits growth of Ewing's sarcoma. Nat. Med. *15*, 750–756. https://doi.org/10.1038/nm.1983.

Florian, S., and Mitchison, T.J. (2016). Anti-microtubule drugs. Methods Mol. Biol. *1413*, 403–421. https://doi.org/10.1007/978-1-4939-3542-0_25.

Han, T., Goralski, M., Capota, E., Padrick, S.B., Kim, J., Xie, Y., and Nijhawan, D. (2016). The antitumor toxin CD437 is a direct inhibitor of DNA polymerase α. Nat. Chem. Biol. *12*, 511–515. https://doi.org/10.1038/nchembio.2082.

Han, T., Goralski, M., Gaskill, N., Capota, E., Kim, J., Ting, T.C., Xie, Y., Williams, N.S., and Nijhawan, D. (2017). Anticancer sulfonamides target splicing by inducing RBM39 degradation via recruitment to DCAF15. Science *356*, eaal3755. https://doi.org/10.1126/science.aal3755.

Han, T., and Nijhawan, D. (2019). Exome sequencing of drug-resistant clones for target identification. Methods Mol. Biol. *1888*, 175–187. https://doi.org/10.1007/978-1-4939-8891-4_10.

Hu, H.M., Zielinska-Kwiatkowska, A., Munro, K., Wilcox, J., Wu, D.Y., Yang, L., and Chansky, H.A. (2008). EWS/FLI1 suppresses retinoblastoma protein function and senescence in Ewing's sarcoma cells. J. Orthop. Res. *26*, 886–893. https://doi.org/10.1002/jor.20597.

Hua, X.H., Genini, D., Gussio, R., Tawatao, R., Shih, H., Kipps, T.J., Carson, D.A., and Leoni, L.M. (2001). Biochemical genetic analysis of indanocine resistance in human leukemia. Cancer Res. *61*, 7248–7254.

Huang, L., Zhai, Y., La, J., Lui, J.W., Moore, S.P.G., Little, E.C., Xiao, S., Haresi, A.J., Brem, C., Bhawan, J., and Lang, D. (2021). Targeting pan-ETS factors inhibits melanoma progression. Cancer Res. *81*, 2071–2085. https://doi.org/10.1158/0008-5472.CAN-19-1668.

Jost, M., Chen, Y., Gilbert, L.A., Horlbeck, M.A., Krenning, L., Menchon, G., Rai, A., Cho, M.Y., Stern, J.J., Prota, A.E., et al. (2017). Combined CRISPRi/a-based chemical genetic screens reveal that rigosertib is a microtubule-destabilizing agent. Mol. Cell *68*, 210–223.e6. https://doi.org/10.1016/j.molcel.2017.09.012.

Jost, M., Chen, Y., Gilbert, L.A., Horlbeck, M.A., Krenning, L., Menchon, G., Rai, A., Cho, M.Y., Stern, J.J., Prota, A.E., et al. (2020). Pharmaceutical-grade rigosertib is a microtubule-destabilizing agent. Mol. Cell *79*, 191–198.e3. https://doi.org/10.1016/j.molcel.2020.06.008.

Kollareddy, M., Sherrard, A., Park, J.H., Szemes, M., Gallacher, K., Melegh, Z., Oltean, S., Michaelis, M., Cinatl, J., Jr., Kaidi, A., and Malik, K. (2017). The small molecule inhibitor YK-4-279 disrupts mitotic progression of neuroblastoma cells, overcomes drug resistance and synergizes with inhibitors of mitosis. Cancer Lett. *403*, 74–85. https://doi.org/10.1016/j.canlet.2017.05.027.

Li, H., and Durbin, R. (2009). Fast and accurate short read alignment with Burrows-Wheeler transform. Bioinformatics *25*, 1754–1760. https://doi.org/10.1093/bioinformatics/btp324.

Lin, A., Giuliano, C.J., Palladino, A., John, K.M., Abramowicz, C., Yuan, M.L., Sausville, E.L., Lukow, D.A., Liu, L., Chait, A.R., et al. (2019a). Off-target toxicity is a common mechanism of action of cancer drugs undergoing clinical trials. Sci. Transl. Med. *11*, eaaw8412. https://doi.org/10.1126/scitranslmed.aaw8412.

Lin, L., Huang, M., Shi, X., Mayakonda, A., Hu, K., Jiang, Y.Y., Guo, X., Chen, L., Pang, B., Doan, N., et al. (2019b). Super-enhancer-associated MEIS1 promotes transcriptional dysregulation in Ewing sarcoma in co-operation with EWS-FLI1. Nucleic Acids Res. *47*, 1255–1267. https://doi.org/10.1093/nar/gky1207.

Ludwig, J.A., Federman, N.C., Anderson, P.M., Macy, M.E., Riedel, R.F., Davis, L.E., Daw, N.C., Wulff, J., Kim, A., Ratan, R., et al. (2021). TK216 for relapsed/refractory Ewing sarcoma: interim phase 1/2 results. J. Clin. Oncol. *39*, 11500. https://doi.org/10.1200/JCO.2021.39.15_suppl.11500.

Ma, C., Tran, J., Li, C., Ganesan, L., Wood, D., and Morrissette, N. (2008). Secondary mutations correct fitness defects in Toxoplasma gondii with dinitroaniline resistance mutations. Genetics *180*, 845–856. https://doi.org/10.1534/genetics.108.092494.

McKenna, A., Hanna, M., Banks, E., Sivachenko, A., Cibulskis, K., Kernytsky, A., Garimella, K., Altshuler, D., Gabriel, S., Daly, M., and DePristo, M.A. (2010). The Genome Analysis Toolkit: a MapReduce framework for analyzing next-generation DNA sequencing data. Genome Res. *20*, 1297–1303. https://doi.org/10.1101/gr.107524.110.

McLoughlin, E.C., and O'Boyle, N.M. (2020). Colchicine-binding site inhibitors from chemistry to clinic: a review. Pharmaceuticals *13*, 8. https://doi.org/10.3390/ph13010008.

Povedano, J.M., Liou, J., Wei, D., Srivatsav, A., Kim, J., Xie, Y., Nijhawan, D., and McFadden, D.G. (2019). Engineering forward genetics into cultured cancer cells for chemical target identification. Cell Chem. Biol. *26*, 1315–1321.e3. https://doi.org/10.1016/j.chembiol.2019.06.006.

Povedano, J.M., Rallabandi, R., Bai, X., Ye, X., Liou, J., Chen, H., Kim, J., Xie, Y., Posner, B., Rice, L., et al. (2020). A multipronged approach establishes covalent modification of beta-tubulin as the mode of action of benzamide anticancer toxins. J. Med. Chem. *63*, 14054–14066. https://doi.org/10.1021/acs.jmedchem.0c01482.

Rahim, S., Minas, T., Hong, S.H., Justvig, S., Celik, H., Kont, Y.S., Han, J., Kallarakal, A.T., Kong, Y., Rudek, M.A., et al. (2014). A small molecule inhibitor of ETV1, YK-4-279, prevents prostate cancer growth and metastasis in a mouse xenograft model. PLoS One *9*, e114260. https://doi.org/10.1371/journal.pone.0114260.

Riggi, N., Suvà, M.L., and Stamenkovic, I. (2021). Ewing's sarcoma. N. Engl. J. Med. *384*, 154–164. https://doi.org/10.1056/NEJMra2028910.

Schibler, M.J., and Huang, B. (1991). The colR4 and colR15 beta-tubulin mutations in Chlamydomonas reinhardtii confer altered sensitivities to microtubule inhibitors and herbicides by enhancing microtubule stability. J. Cell Biol. *113*, 605–614. https://doi.org/10.1083/jcb.113.3.605.

Selvanathan, S.P., Graham, G.T., Erkizan, H.V., Dirksen, U., Natarajan, T.G., Dakic, A., Yu, S., Liu, X., Paulsen, M.T., Ljungman, M.E., et al. (2015). Oncogenic fusion protein EWS-FLI1 is a network hub that regulates alternative splicing. Proc. Natl. Acad. Sci. USA *112*, E1307–E1316. https://doi.org/10.1073/pnas.1500536112.

Spriano, F., Chung, E.Y.L., Gaudio, E., Tarantelli, C., Cascione, L., Napoli, S., Jessen, K., Carrassa, L., Priebe, V., Sartori, G., et al. (2019). The ETS inhibitors YK-4-279 and TK-216 are novel antilymphoma agents. Clin. Cancer Res. *25*, 5167–5176. https://doi.org/10.1158/1078-0432.CCR-18-2718.

Wacker, S.A., Houghtaling, B.R., Elemento, O., and Kapoor, T.M. (2012). Using transcriptome sequencing to identify mechanisms of drug action and resistance. Nat. Chem. Biol. *8*, 235–237. https://doi.org/10.1038/nchembio.779.

Wordeman, L., and Vicente, J.J. (2021). Microtubule targeting agents in Disease: classic drugs, novel roles. Cancers *13*, 5650. https://doi.org/10.3390/cancers13225650.

Xiang, Q., Zhen, Z., Deng, D.Y., Wang, J., Chen, Y., Li, J., Zhang, Y., Wang, F., Chen, N., Chen, H., and Chen, Y. (2015). Tivantinib induces G2/M arrest and apoptosis by disrupting tubulin polymerization in hepatocellular carcinoma. J. Exp. Clin. Cancer Res. *34*, 118. https://doi.org/10.1186/s13046-015-0238-2.

Xue, J., Li, S., Shi, P., Chen, M., Yu, S., Hong, S., Li, Y., Liu, R., and Xiao, H. (2021). The ETS inhibitor YK-4-279 suppresses thyroid cancer progression independent of TERT promoter mutations. Front. Oncol. *11*, 649323. https://doi.org/10.3389/fonc.2021.649323.

Zöllner, S.K., Selvanathan, S.P., Graham, G.T., Commins, R.M.T., Hong, S.H., Moseley, E., Parks, S., Haladyna, J.N., Erkizan, H.V., Dirksen, U., et al. (2017). Inhibition of the oncogenic fusion protein EWS-FLI1 causes G2-M cell cycle arrest and enhanced vincristine sensitivity in Ewing's sarcoma. Sci. Signal. *10*, eaam8429. https://doi.org/10.1126/scisignal.aam8429.

STAR★METHODS

KEY RESOURCES TABLE

REAGENT or RESOURCE	SOURCE	IDENTIFIER
**Chemicals, peptides, and recombinant proteins**		
Etoposide	Sigma- Aldrich	Cat# E1383; CAS ID: 33419-42-0
CD437	Sigma-Aldrich	Cat# C5865; CAS ID: 125316-60-1
MLN4924	ApexBio	Cat# B1036; CAS ID: 905579-51-3
Rigosertib	Cayman Chemicals	Cat# 15553; CAS ID: 1225497-78-8
Tivantinib (ARQ 197)	Cayman Chemicals	Cat# 17135; CAS ID: 905854-02-6
Colchicine	Sigma-Aldrich	Cat# C9754; CAS ID: 64-86-8
Vincristine	Cayman Chemicals	Cat# 11764; CAS ID: 2068-78-2
YK-4-279	Selleck Chemicals	Cat# S7679; CAS ID: 1037184-44-3
TK216 (ONCT-216)	MedChem Express	Cat# HY-122903; CAS ID: 1903783-48-1
**Critical commercial assays**		
CellTiter-Glo luminescent cell viability assay	Promega	Cat# G7571
Tubulin Tracker Deep Red	ThermoFisher	Cat# T34077
SF Cell Line 4D-Nucleofector™ X Kit L	LONZA	Cat# V4XC-3024
**Deposited data**		
WES data for samples TK216 clones	This paper	SRA: PRJNA770630 (https://www.ncbi.nlm.nih.gov/sra/PRJNA770630)
RNA sequencing of A673 cells data	(Lin et al., 2019a, 2019b)	GEO: GSE117485 (https://www.ncbi.nlm.nih.gov/geo/query/acc.cgi?acc=GSM3301789)
**Experimental models: Cell lines**		
Ewing sarcoma A673 cell line	ATCC	ATCC® CRL-1598™
**Oligonucleotides**		
Oligonucleotides for KI TUBA1B mutations, see Table S1	This paper	N/A
Primers for sequencing TUBA1B mutations, see Table S1	This paper	N/A
Oligonucleotides for KI TUBB4B mutations, see Table S1	This paper	N/A
Primers for sequencing TUBB4B mutations, see Table S1	This paper	N/A
**Software and algorithms**		
Trim Galore	N/A	https://www.bioinformatics.babraham.ac.uk/projects/trim_galore/
Genome Analysis Toolkit (GATK, 4.1.4.0)	(DePristo et al., 2011; McKenna et al., 2010)	N/A
Burrows-Wheeler Aligner (BWA, v0.7.17)	(Li and Durbin, 2009)	N/A
Picard (2.21.3)	N/A	https://broadinstitute.github.io/picard
GATK HaplotypeCaller	N/A	N/A
Annomen	N/A	https://github.com/jiwoongbio/Annomen

RESOURCE AVAILABILITY

Lead contact

Further information and requests for resources and reagents should be directed to and will be fulfilled by the Lead Contact, David McFadden (david.mcfadden@utsouthwestern.edu).

Material availability

This study did not generate new unique reagents.

Data and code availability

- WES data for samples TK216 clones have been deposited at Sequence Read Archive (SRA) and are publically available as of the date of publication. RNA sequencing of A673 cells data used to identify the expression pattern of different of α- and β-tubulins are publicaly available at GEO. Accession numbers are listed in the Key resources table.
- This paper does not report original code.
- Any additional information required to reanalyze the data reported in this paper is available from the Lead contact upon request.

EXPERIMENTAL MODEL AND SUBJECT DETAILS

Ewing sarcoma A673 cell lines were cultured at 37°C and 5% CO_2 in RPMI (R8758, Sigma-Aldrich) and supplemented with 10% FBS (#35-150-CV, Corning), 2 mM L-glutamine (G7513, Sigma-Aldrich), and penicillin/streptomycin (P0781, Sigma-Aldrich). Cells were passaged using trypsin (T4049, Sigma-Aldrich) every 3-4 days. Parental A673 cell lines are derived from a female subject and were authenticated by STR profiling.

METHOD DETAILS

Compounds

Etoposide was purchased from Sigma-Aldrich (#E1383-100MG). CD437 was purchased from Sigma-Aldrich (#C5865). MLN4924 was purchased from ApexBio (#B1036). Rigosertib was purchased from Cayman Chemical (#15553). Tivantinib (ARQ 197) was purchased from Cayman Chemical (#17135). Colchicine was purchased from Sigma-Aldrich (#C9754-100MG). Vincristine was purchased from Cayman Chemical (#11764). YK-4-279 was purchased from Selleck Chemicals (#S7679). TK216 was purchased from MedChem Express (#HY-122903). TK216 enantiomers were separated and purified by Lotus Separations. SFC (supercritical fluid chromatography) separation of 29 mg racemic TK216 yielded 14 mg of (+)-TK216 and 14 mg of (−)-TK216. The separation method used was: AS-H (2 × 25 cm), 35% ethanol/CO_2 (100 bar), 50 mL/min, 220 nm. Injected volume 1 mL, 2 mg/mL methanol:DCM. YK-4-279, TK216, and both TK216 enantiomers exhibited between 98 and 99% purity as determined by LC-MS analysis performed on an Agilent 1290 HPLC system using an Eclipse XDB-C18 column (46 × 150 mm, 5 μm); Agilent) that was coupled to an Agilent 6130 mass spectrometer run in ESI mode in both positive and negative ionization with a scan range of 100-1,100 *m/z*. Liquid chromatography was carried out at a flow rate of 0.5 mL/min at 20°C with a 5 μL injection volume, using the gradient elution with aqueous acetonitrile containing 0.1% formic acid. The gradient was adjusted based on the different polarity of different compounds. All compounds were diluted in DMSO (Sigma-Aldrich, D650-100ML). HPLC chromatograms for all compounds used in the study shown in Data S1 and S2.

Forward genetic screen of TK216

Previously described mismatch-repair deficient EWS cells, A673-M1, were utilized (Povedano et al., 2019). We first identified the concentration of TK216 that killed 100% of MMR-deficient and -proficient A673 cells after 1 week of compound exposure (IC_{100}^{1wk}). IC_{100}^{1wk} determination for TK216 was performed in a 12-well plate seeding 25,000 cells per well. After 24h, TK216 was dispensed using TECAN D300e setting up a minimum concentration of IC_{50}^{72h} and a maximum concentration of IC_{100}^{72h}. Media and TK216 were replenished after 3-4 days. After 7 days, cell viability was determined visually to determine IC_{100}^{1wk}. Then, A673-M1 cells and A673 parental cells were plated in 5 × 10cm plates for each cell line (1 million cells per plate). The following day, TK216 was added at 3 different concentrations: IC_{100}^{1wk} ÷ 1.25 (0.78 μM), IC_{100}^{1wk} (0.98 μM), and IC_{100}^{1wk} x 1.25 (1.22 μM) to the plates. Media with TK216 was replenished every 3–4 days over the course of 2 weeks. Surviving clones were picked and expanded.

Whole exome sequencing analysis

Trim Galore (https://www.bioinformatics.babraham.ac.uk/projects/trim_galore/) was used for quality and adapter trimming. The human reference genome sequence and gene annotation data, hg38, were downloaded from Illumina iGenomes (https://support.illumina.com/sequencing/sequencing_software/igenome.html). The sequencing reads were aligned to the genome sequence using Burrows-Wheeler Aligner (BWA, v0.7.17) (Li and Durbin, 2009). Picard (2.21.3) (https://broadinstitute.github.io/picard) was used to remove PCR duplicates and Genome Analysis Toolkit (GATK, 4.1.4.0) (DePristo et al., 2011; McKenna et al., 2010) was used to recalibrate base qualities. Calling variants and genotyping were performed using GATK HaplotypeCaller and the variant calls were filtered by applying the following criteria: QD (Variant Confidence/Quality by Depth) < 2, FS (Phred-scaled p-value using Fisher's exact test to detect strand bias) > 60, MQ (RMS Mapping Quality) < 40, DP (Approximate read depth) < 3, GQ (Geno-type Quality) < 7. Custom Perl scripts (https://github.com/jiwoongbio/Annomen) were used to annotate variants with human transcripts, proteins, and variations (RefSeq and dbSNP build 151) and calculate variant allele frequencies.

We defined acquired somatic mutations for each A673-M1 TK216-resistant clones by VAF >0.2 and VAF <0.01 for the parental A673-M1 cell line and VAF <0.05 for previously reported MLN-resistant clones (Povedano et al., 2019). Non-coding mutations were excluded from the analysis.

Introduction of tubulin mutations

We performed homology-directed repair using Alt-R CRISPR-Cas9 System and ultramer oligo from Integrated DNA Technologies (IDT) in A673 EWS cells. To prepare the gRNA complex for *TUBA1B*^{G142}, *TUBA1B*^{D47}, and *TUBB4B*^{K350} we combined 10 μL of Alt-R CRISPR-Cas9 crRNA (100 μM) (*TUBA1B*^{G142} sequence: 5′-UUCUUGGUUUUCCACAGCUUGUUUUAGAGCUAUGCU-3′; *TUBA1B*^{D47} sequence: 5′-CUCACUGAAGAAGGUGUUGAGUUUUAGAGCUAUGCU-3′; *TUBB4B*^{K350} sequence: 5′-GAUCCCCAACAAUGUGAAAAGUUUUAGAGCUAUGCU-3′) and 10 μL Alt-R CRISPR-Cas9 tracrRNA (100 μM). The mixture was heated to 95°C for 5 min, then allowed to slowly cool to room temperature. Ribonucleoprotein complex with Cas9 was formed by combining 3 μL of gRNA complex with 2 μL Alt-R Cas9 enzyme and incubating at RT for 10-20 min. Two million A673 cells were resuspended in 120 μL of SF Cell Line 4D-NucleofectorTM X Kit L (LONZA, Cat. #: V4XC-3024). The transfection mix was prepared using: 15 μL of RNP complex, 3.6 μL of 100 μM Ultramer ssODN donor (*TUBA1B*^{G142S} sequence: 5′-A*C*C AGT GCA CCG GTC TTC AGG GCT TCT TGG TTT TCC ACA GCT TTA GTG GGG GAA CTG GTT CTG GGT TCA CCT CCC TGC TCA TG*G*A-3′; *TUBA1B*^{G142A} sequence: 5′-A*C*C AGT GCA CCG GTC TTC AGG GCT TCT TGG TTT TCC ACA GCT TTG CTG GGG GAA CTG GTT CTG GGT TCA CCT CCC TGC TCA TG G*A*A-3′; *TUBA1B*^{D47H} sequence: T*G*G CCA GAT GCC AAG TGA CAA GAC CAT TGG GGG AGG AGA TGC CTC CTT CAA CAC ATT CTT CAG TGA GAC GGG CGC TGG CAA GCA CGT GCC CCG GGC T*G*T; *TUBA1B*^{D47G} sequence: T*G*G CCA GAT GCC AAG TGA CAA GAC CAT TGG GGG AGG AGA TCA CTC CTT CAA CAC ATT CTT CAG TGA GAC GGG CGC TGG CAA GCA CGT GCC CCG GGC T*G*T; *TUBB4B*^{K350T} sequence: 5′- T*C*C AAA ACA AAA ACA GCA GCT ATT TTG TTG AGT GGA TCC CCA ACA ATG TGA CAA CTG CTG TCT GTG ACA TCC CAC CTC GGG GGC TAA AAA TGT CCG CCA CCT TC*A*T-3′) and added to 60 μL of previously prepared cell suspension. Nucleofection was performed using 4D-Nucleofector core unit from LONZA. Cells were plated into one well of a 6-w plate after nucleofection in 2 mL of RPMI (10% FBS). After one week, sham cells or ssODN (*TUBA1B*$^{G142\ S/A}$) cells were plated into 10 cm dishes. Each sample was plated into three 10cm dishes using one million cells per dish. *TUBA1B*$^{G142S/A}$ cells were treated with TK216 at concentrations: 0.75/1/1.25 μM. Media and small molecules were replenished every 3–4 days over the course of 2 weeks followed by expansion in media without compound for 1 week. A673-*TUBA1B*$^{G142\ S/A}$ and -*TUBA1B*$^{D47\ H/G}$ cells were stained with crystal violet staining solution prepared with 1% (weight/volume ratio) crystal violet from Sigma-Aldrich (#C6158-50G) in 10% ethanol. After TK216 selection, one dish with A673-*TUBA1B*^{G142S}, -*TUBA1B*^{G142A}, and -*TUBA1B*^{D47H} cells were expanded to performed DRC to validate compound resistance. After TK216 selections, emerging clones from A673-*TUBB4B*^{K350T} cells were picked and expanded.

Cytotoxic assay

EWS A673 cells were seeded in duplicate in 96-well plates, 3,000 cells and 200 μL of RPMI media (10% FBS) per well. After overnight incubation, compounds were dispensed using a D300e Digital Dispenser (TECAN) in 15-point dose response manner using a maximum and minimum concentration of 50 μM and 1.58 nM, respectively. Cell viability was assessed after 72 h using CellTiter-Glo luminescent cell viability assay (Promega, #G7571). The CellTiter-Glo reagent was diluted by adding PBS-Triton-X (1%) (1:1 ratio). Each value was normalized to cells treated with DMSO, and the IC50 values were calculated using GraphPad Prism software.

MT polymerization assay

We used cycled-tubulin purchased from PurSolutions (Cat. #: 032,005). MT polymerization occurs spontaneously upon incubation of cycled-tubulin in PEM buffer with GTP at 37 Celsius. Each condition was performed in triplicate from a 384-well plate. First, the 384-well plate was prewarmed at 37 Celsius using plate reader Synergy2 (Biotek). Master mix containing cycled-tubulin, PEM buffer and GTP was prepared for the samples analyzed in an Eppendorf tube: 2.5 μg of cycled-tubulin (20 mg/mL), (5X) PEM buffer (400 mM PIPES, 5 mM EGTA, 5 mM $MgCl_2$, pH = 6.8), DTT (1 mM), GTP (1 mM), DMSO (3%), and ddH_2O up to 30 μL per reaction and incubateed on ice 3-5 min. After incubation, 30 μL of the master mix was added per well in a 384-well plate. Immediately after compounds were added at desired concentration using a D300e Digital Dispenser (TECAN). Absorbance at 340 nm was measured immediately after every 15 s for 20 min.

Cell-based tubulin probe competition assay

Human EWS cells, A673, were seeded in 12-w plates (0.5 million cells per well) with 1 mL RPMI media (10% FBS) each well. Small molecules were dispensed 24-h later at increasing concentrations using a D300e Digital Dispenser (TECAN) in 10-point dose response manner using a maximum and minimum concentration of 50 μM and 100 nM. Following 30 min incubation at 37°C in 5% CO_2, tubulin covalent probe was dispensed at 5 μM in 11 wells. After 30 min incubation, cells were washed gently with PBS and then lysed in 1% SDS Buffer A (50 mM HEPES pH 7.4, 10 mM KCl, 2 mM $MgCl_2$), freshly supplemented with 1:10,000 benzonase (Sigma-Aldrich). After incubation, copper-mediated click chemistry with a fluorescent azide was performed. Covalently modified β-tubulin was visualized by SDS-PAGE and scanning gels for fluorescence (Povedano et al., 2020).

Microscopy of microtubules

0.5*10^6 A673 and A673-*TUBA1B*^{G142A} EWS cells were plated per condition using RPMI (10% FBS) onto a 35mm Petri glass bottom dish (MatTek, Part #: P35G-1.5-14-C). These dishes were previously coated with gelatin for 30 min to increase cell attachment. Following 24 h, DMSO, (−)-TK216 (1 μM), or colchicine (50 nM) were added to both EWS cells. After 20 h of incubation, media was exchanged with phenol red-free media (10% FBS). Tubulin Tracker Deep Red (ThermoFisher, Cat. #: T34077) was diluted as

described by manufacturer and added to the media for 30 min. Cells were rinsed three times with phenol red-free media including 1X Probenecid (ThermoFisher, Cat. #: T34077) and Hoechst 33342 (Invitrogen, Ref: H3570) as described by manufacturer.

QUANTIFICATION AND STATISTICAL ANALYSIS

Data were analyzed using Prism 9 by GraphPad. Dose-response curves were fitting in Figures 1B, 1C, 1H–1M, 2A–2I, S1A, S2D–S2M, S3B, and S3F, to calculate the IC_{50}. Hill coefficients and standard errors were done using Log [inhibitor] vs Normalized response. Quantitative data are presented as mean.

Cell Chemical Biology, Volume *29*

Supplemental information

TK216 targets microtubules in Ewing sarcoma cells

Juan Manuel Povedano, Vicky Li, Katherine E. Lake, Xin Bai, Rameshu Rallabandi, Jiwoong Kim, Yang Xie, Jef K. De Brabander, and David G. McFadden

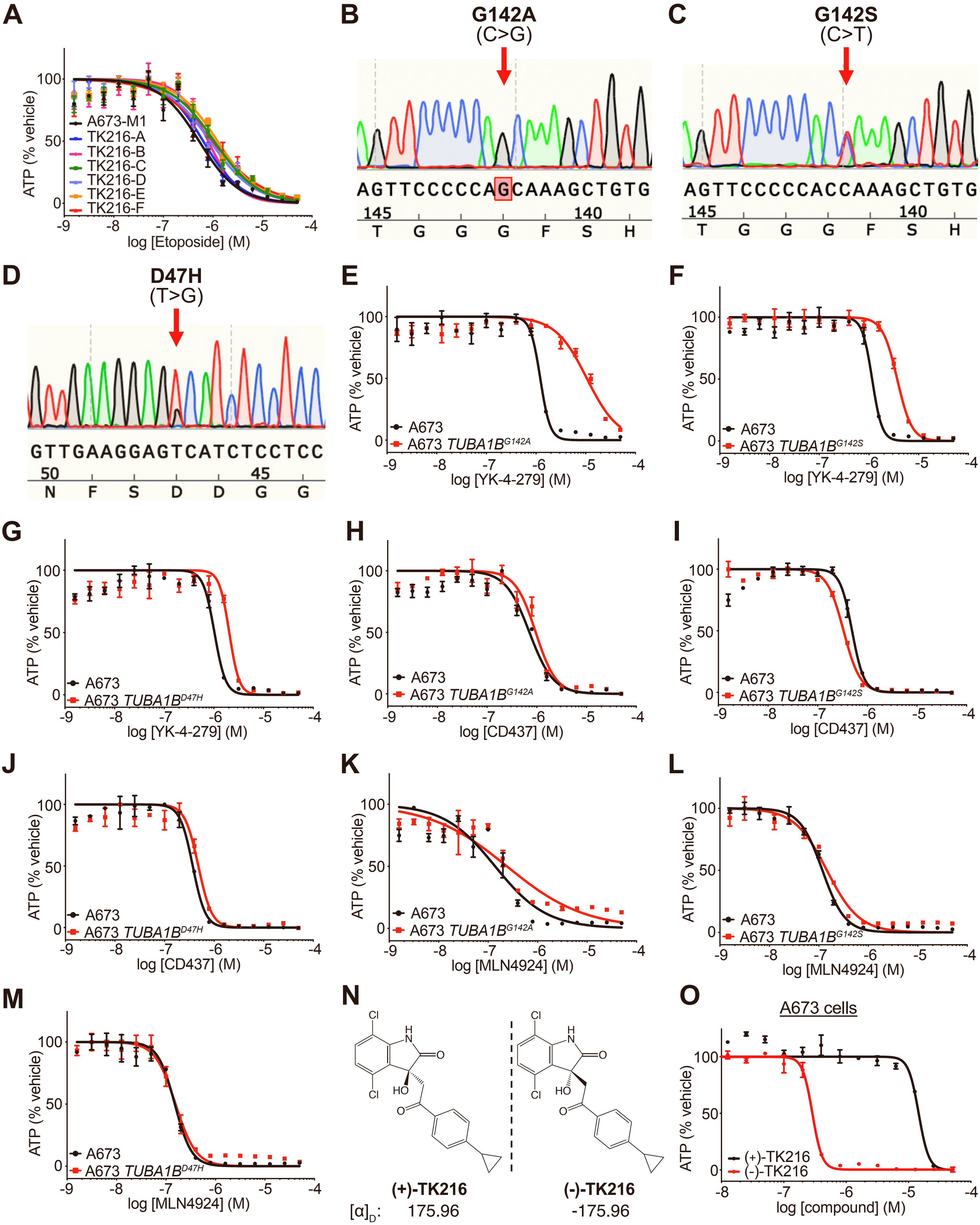

Figure S1, related to Figure 1. (A) Dose-response curve for etoposide against A673-M1, Msh2-null EWS cells, and TK216 resistant clones. (B,C,D) Sanger sequencing traces for *TUBA1B*^{G142A}, *TUBA1B*^{G142S}, and *TUBA1B*^{D47H} mutations in EWS cells. (E,H,K) Dose-response curves for, YK-4-279 (E), CD437 (H), and MLN4924 (K) against EWS cells harboring *TUBA1B*^{G142A} mutation. (F,I,L) Dose-response curves for, YK-4-279 (F), CD437 (I), and MLN4924 (L) against EWS cells harboring *TUBA1B*^{G142S} mutation. (G,J,M) Dose-response curves for, YK-4-279 (G), CD437 (J), and MLN4924 (M) against EWS cells harboring *TUBA1B*^{D47H} mutation. (N) Chemical structure and specific rotation ($[\alpha]_D$) of each TK216 enantiomer. (O) Dose-response curve for (-)-TK216 and (+)-TK216, against EWS A673 cells.

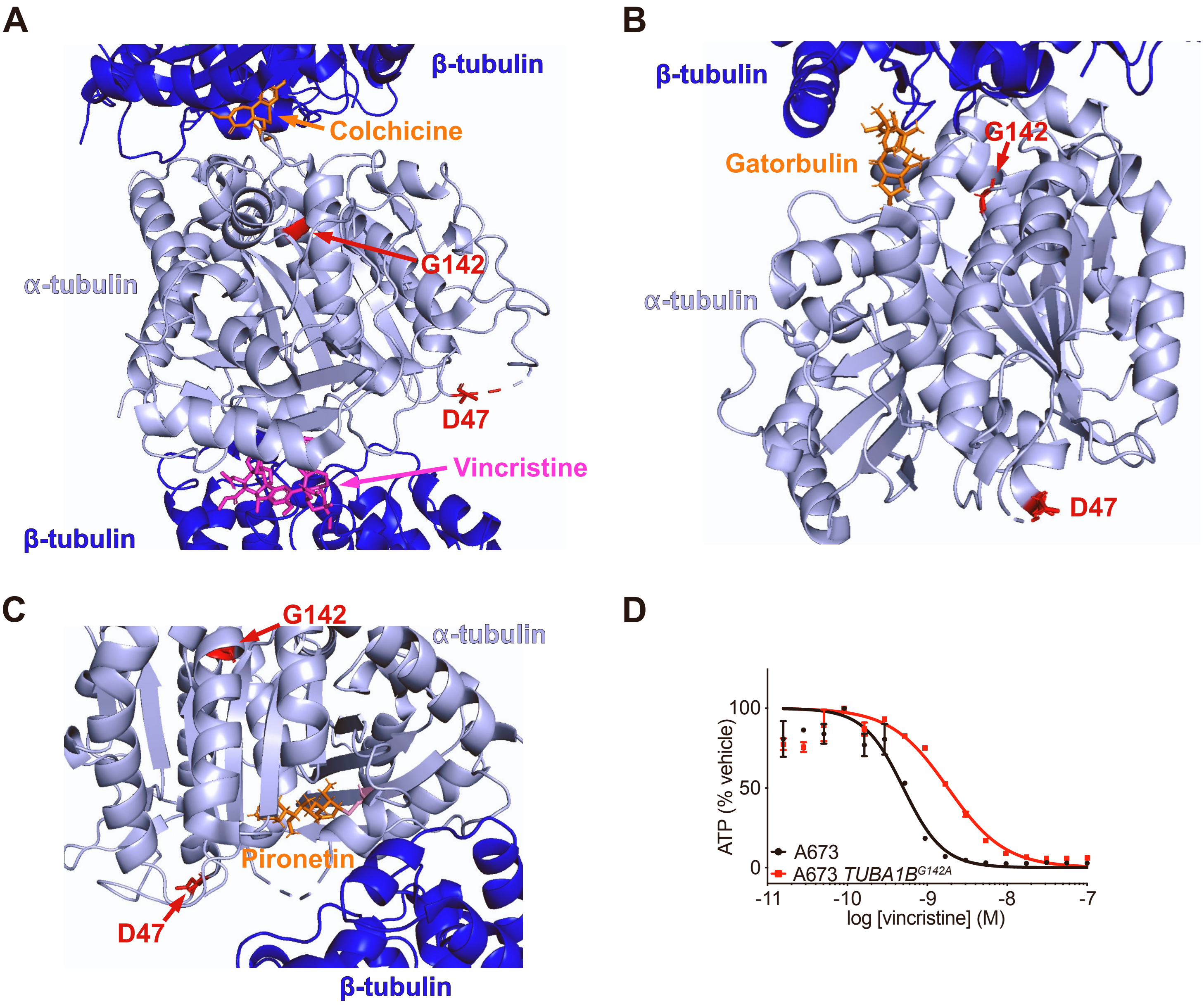

Figure S2, related to Figures 1 and 2. (A-C) Co-crystal structure of α-/β-tubulin with colchicine and vincristine (PDB: 1Z2B) (A), gatorbulin (PDB: 7alr) (B), and pironetin (PDB: 5la6) (C). (D) Dose-response curve for vincristine against EWS cells harboring *TUBA1B*^{G142A} mutation.

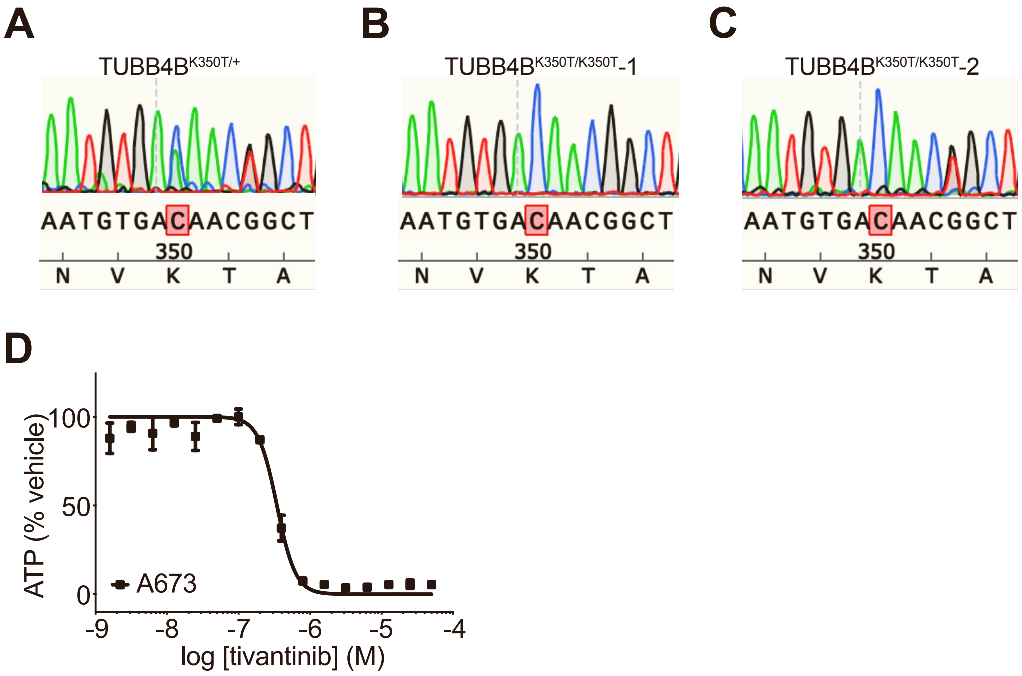

Figure S3, related to Figures 2 and 4. (A-C) Sequencing traces for *TUBB4B*^{K350T} mutation in three independent clones of EWS cells. (D) Dose-response curve for Tivantinib against EWS A673 cells.

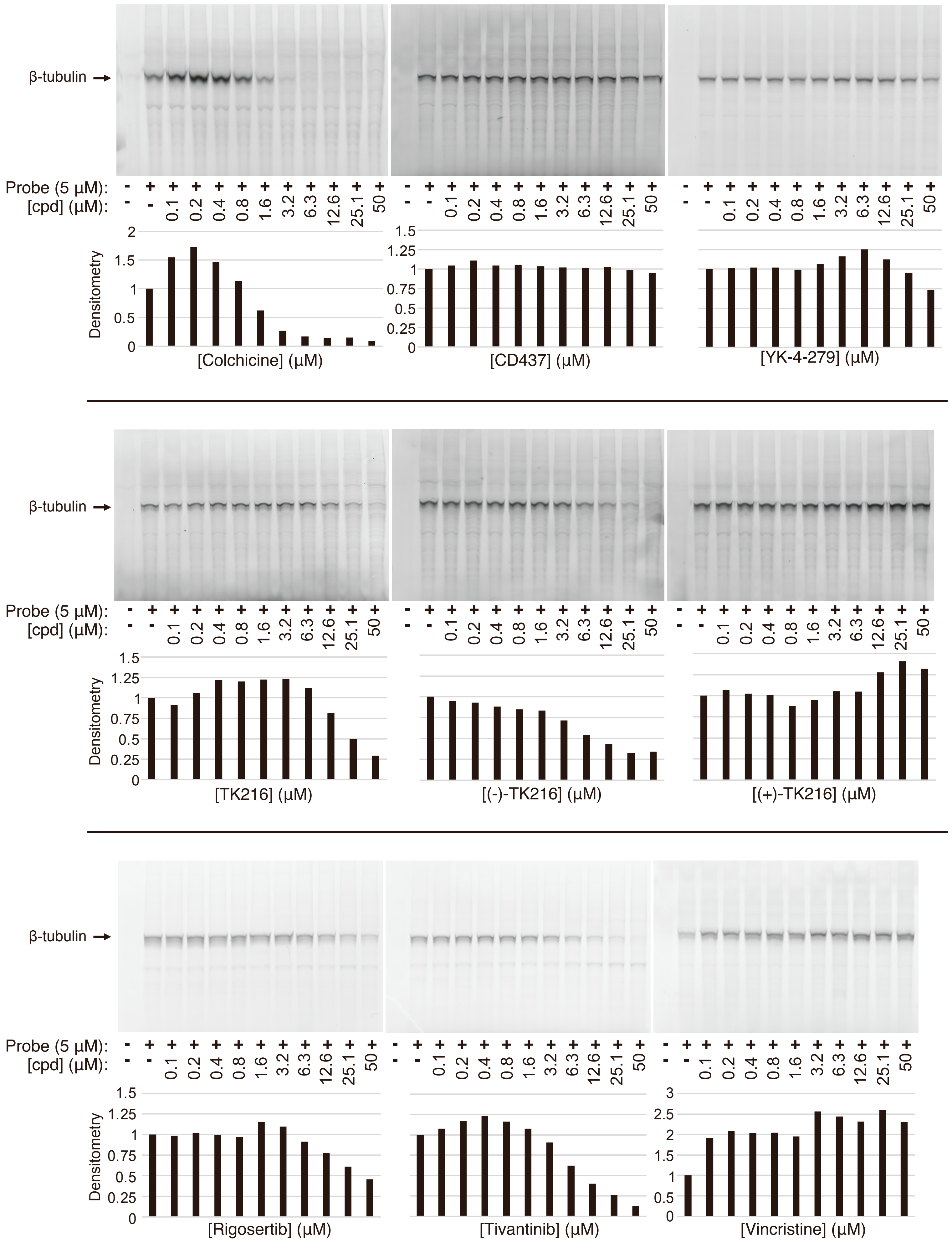

Figure S4, related to Figure 4. Full gel images of cell-based competition assay using covalent β-tubulin probe to assess tubulin binding in EWS cells by compounds, colchicine, CD437, YK-4-279, TK216, (-)-TK216, (+)-TK216, rigosertib, tivantinib, and vincristine. Densitometry graphs under gels were generated using ImageJ to measure intensity of β-tubulin band. Densitometry data were normalized to the control no competitor plus probe lane.

A

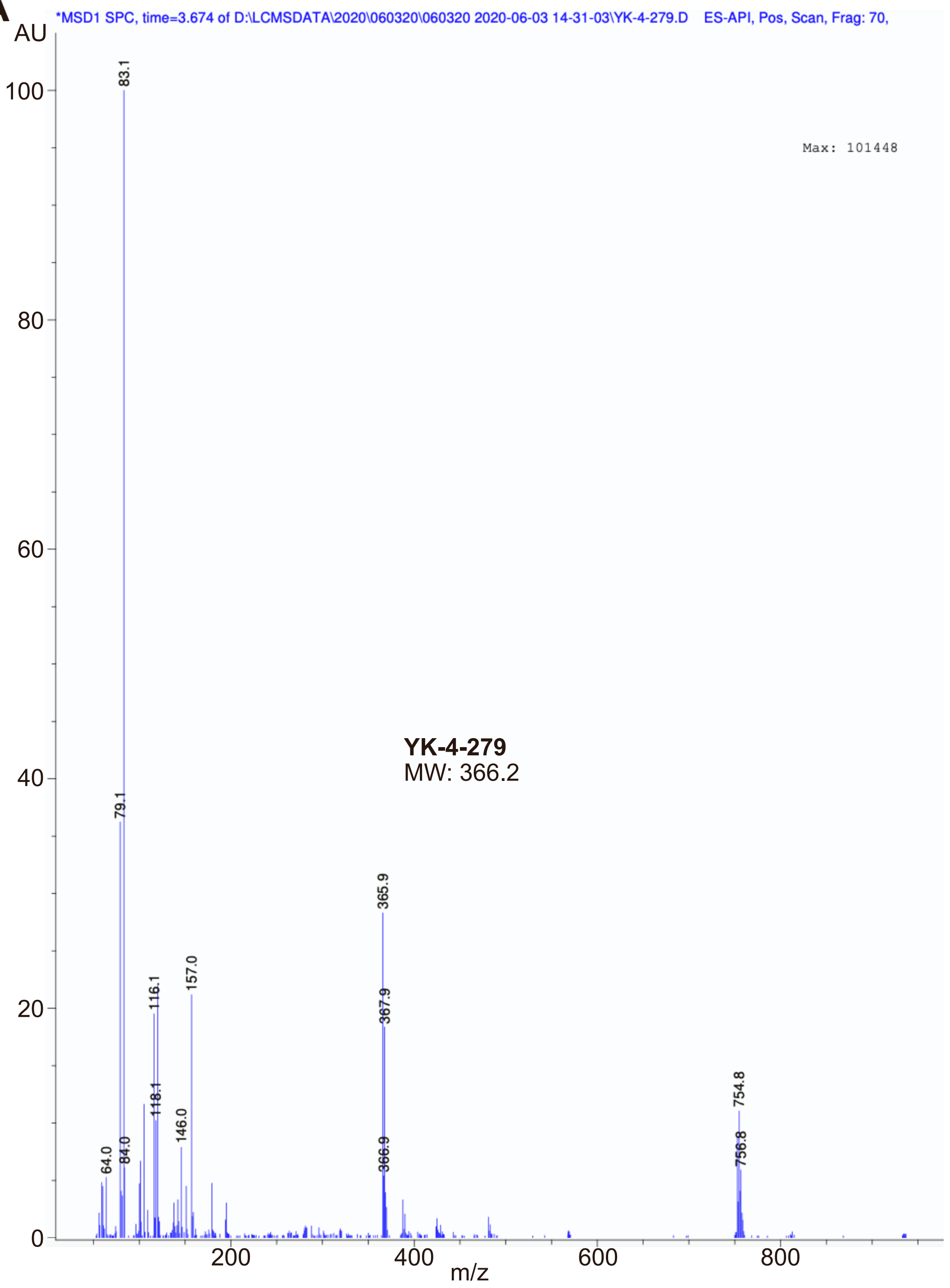

B

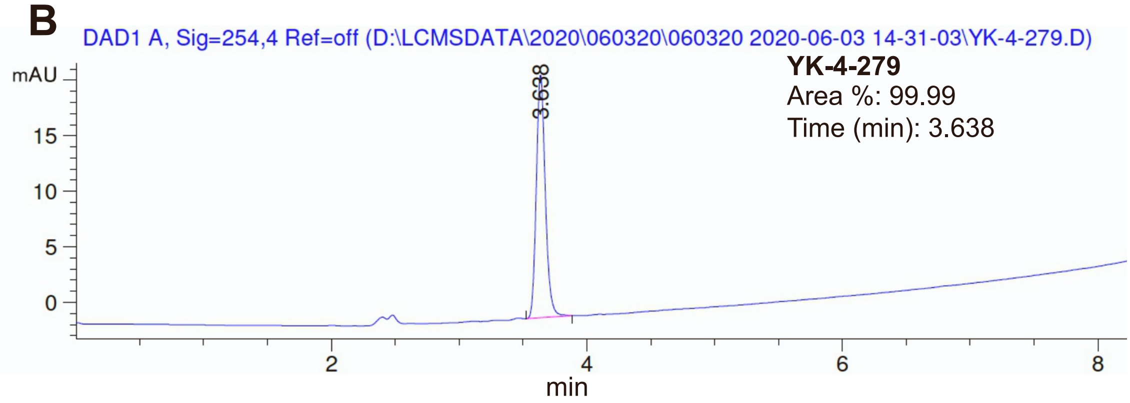

Data S1. HPLC (A) and HRMS (B) data of YK-4-279. Related to Figures 2, 3, and 4

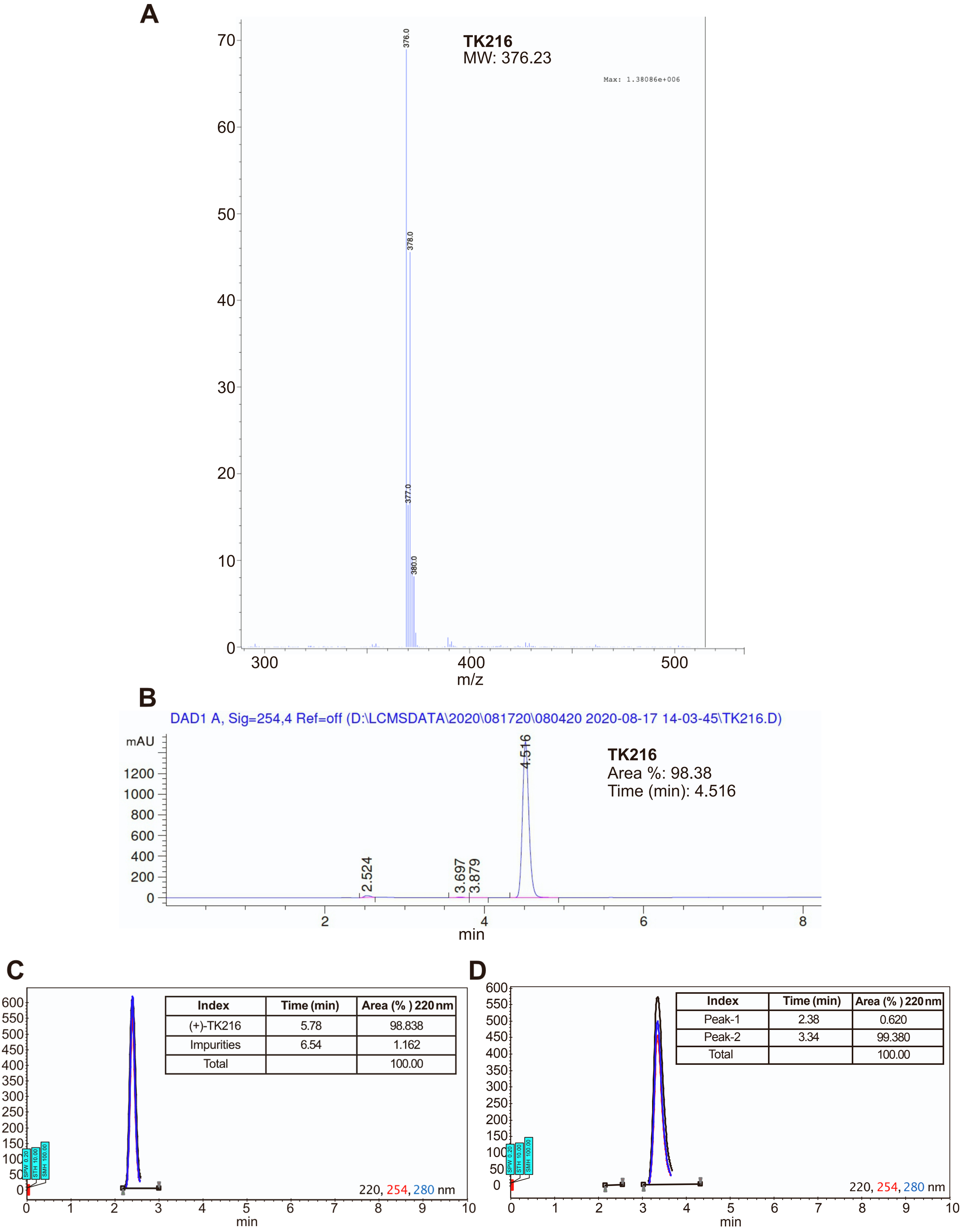

Index	Time (min)	Area (%) 220 nm
(+)-TK216	5.78	98.838
Impurities	6.54	1.162
Total		100.00

Index	Time (min)	Area (%) 220 nm
Peak-1	2.38	0.620
Peak-2	3.34	99.380
Total		100.00

Data S2. HRMS (A) and HPLC (B) data of TK216, and HPLC data for (+)-TK216 (C) and (-)-TK216 (D). Related to Figures 1, 2, 3, and 4.
